# Tuning superposition of multiple Fano interferences for efficient plasmon resonance energy transfer to the assembled molecules

**DOI:** 10.1038/s41377-026-02381-8

**Published:** 2026-09-09

**Authors:** Yishuang Wang, Xueqing Sang, Zhen-Long Dou, Qin-Xing Zhou, Zhiyu Zhao, Da-Jie Yang, Yan Zhang, Li Zhou, Xiaoguang Li, Qu-Quan Wang

**Affiliations:** 1 Department of Physics, State Key Laboratory of Quantum Functional Materials, and Guangdong Basic Research Center of Excellence for Quantum Science, Southern University of Science and Technology, Shenzhen, 518055 China; 2 Key Laboratory of Artificial Micro- and Nano-Structures of the Ministry of Education, Hubei Nuclear Solid Physics Key Laboratory, School of Physics and Technology, Wuhan University, Wuhan, 430072 China; 3 School of Mathematics and Physics, North China Electric Power University, Beijing, 102206 China; 4 Quantum Science Center of Guangdong–Hong Kong–Macao Greater Bay Area (Guangdong), Shenzhen, 518045 China; 5 Institute for Advanced Study, Shenzhen University, Shenzhen, 518060 China; 6 Guangdong Provincial Key Laboratory of Advanced Thermoelectric Materials and Device Physics, Southern University of Science and Technology, Shenzhen, 518055 China

**Keywords:** Nanophotonics and plasmonics, Nanoparticles, Nonlinear optics, Ultrafast photonics

## Abstract

Plasmon–exciton Fano interference provides an efficient channel for coherent energy transfer, and the plasmon resonance energy transfer (PRET) peak is usually observed around the Fano dip at the exciton resonance. In this work, we achieve tunable multiple Fano interferences and multichannel PRET by strategically coupling five excitonic resonances (labeled as M, D_h_, D_j_, H, and J) of assembled IR806 molecules with Au nanobipyramids and nanorods (AuNBPs and AuNRs, respectively). Notably, owing to orientation-dependent molecule–plasmon coupling in the IR806/AuNBP hybrids, we observe two PRET peaks with opposite signs of Fano factor q. One PRET peak remains fixed near the J-aggregate exciton resonance at ~900 nm, whereas the other is located around the plasmon resonance and tuned from 710 to 920 nm. The total PRET reaches a maximum when the two PRET peaks are tuned into spectral overlap. Furthermore, our newly developed semiclassical model quantitatively reproduces these features, which reveals that PRET enhancement arises from the superposition of multiple Fano interferences. These findings provide a novel strategy to enhance the efficiency and tunability of the PRET, opening avenues for advanced applications in ultrafast optical information processing and highly sensitive photodetectors.

## Abstract (web summary)

By tuning the superposition of multiple Fano interferences in IR806/Au hybrids, we achieve spectral overlap of two plasmon resonance energy transfer (PRET) peaks, maximizing energy transfer to assembled IR806 molecules.

## Graphical abstract

**Figure:**
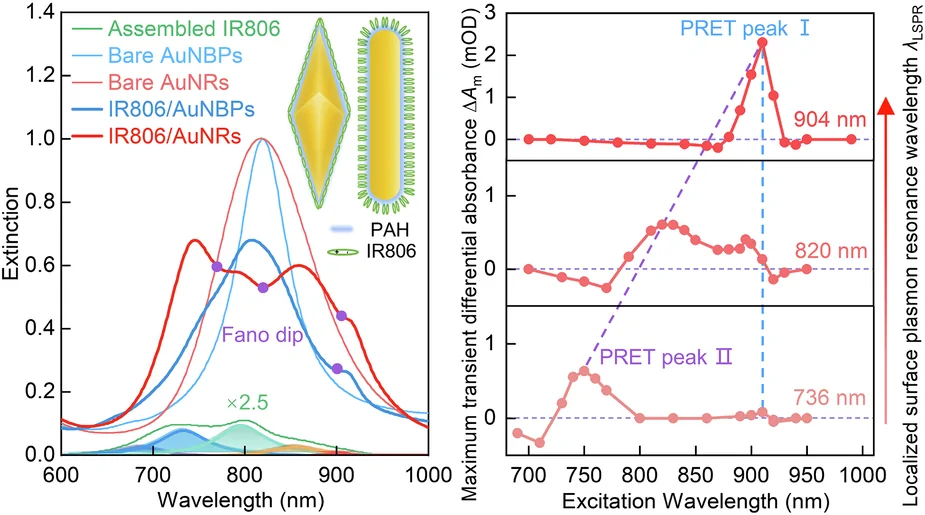

## Introduction

Precise control of light–matter interactions at the nanoscale remains a fundamental challenge in modern photonics^1–3^. Molecule–plasmon hybrids, which combine the unique optical properties of plasmonic nanostructures with the tunable electronic responses of molecular excitons, offer a powerful strategy for overcoming this challenge^4,5^. Extensive research has revealed various underlying physical mechanisms in such hybrid systems, including plasmon-induced charge transfer^6^, ballistic hot-electron injection^7^, plasmon-driven hot-electron transfer^8–10^, and excitation energy transfer^11–13^. Energy transfer from excited plasmon into diverse pathways is of central importance for enhancing Raman scattering^14^, photoluminescence^15–17^, photocurrents^18^, and photocatalysis^19,20^. Among these concerns, plasmon resonance energy transfer (PRET) mediated by Fano interference^21,22^ has attracted considerable attention as an efficient mechanism for enabling coherent energy transfer between plasmons and excitons^23–25^.

Two of the most intriguing manifestations of plasmon–exciton coupling are Fano interference and Rabi splitting^26–30^. Fano interference with a moderate coupling strength is usually characterized by a Fano dip in the hybridized extinction spectrum when the quality factor Q of the plasmon resonance is much lower than that of the excitons^31–33^. The PRET between plasmons and excitons can significantly increase the photocatalytic reaction efficiency, photovoltaic power conversion efficiency, and quantum efficiency of optoelectronic devices. Notably, plasmons predominantly transfer energy via nonradiative channels. This mechanism not only effectively suppresses interfacial charge recombination losses but also prevents plasmon energy dissipation associated with phase mismatch during hot electron relaxation, thereby providing fundamental insights into the design of low-loss photonic energy conversion devices^34,35^. Recently, a hot electron super-generator combining Fano interference with the antenna effect pushes the limits of quantum effects in plasmonics^36^. Ultrafast PRET from plasmon to exciton usually occurs around the position of the Fano dip. Therefore, it is limited to a narrow wavelength region around the exciton resonance and has poor tunability with plasmon resonances^11,37^. Additionally, when the quality factor Q of plasmon becomes comparable to that of exciton, the destructive Fano dip of plasmon–exciton hybrids is no longer prominent, which makes PRET difficult to evaluate from the hybridized extinction spectrum. The tunable spectral response and ultrafast dynamics of the PRET are critically important for improving energy transfer efficiency and better understanding the physical mechanism of coherent and following decoherent processes, but they remain unclear.

In this work, we systematically investigate multiple hybridizations of assembled IR806 molecules (five excitonic states) coupled with the longitudinal surface plasmon resonance (LSPR) of Au nanobipyramids (AuNBPs) in comparison to hybrids formed with Au nanorods (AuNRs). We further explored the nonlinear optical responses induced by PRET in those hybrids. Owing to the distinct orientations of the IR806 molecules on the AuNRs and AuNBPs, J-aggregate excitons dominate multiple Fano interferences in the IR806/AuNBP hybrids, whereas D_j_ and D_h_ excitons are responsible for the interference in the IR806/AuNR hybrids. By adjusting the plasmon wavelength ${\lambda }_{{\rm{LSPR}}}$ of the AuNBPs from 696 to 942 nm, the main peak wavelength ${\lambda }_{{\rm{PRET}}}$ of the reverse saturable absorption (RSA) induced by the PRET of the hybrids can be tuned from 710 to 920 nm. The maximum peak intensity of RSA ($\Delta {A}_{{\rm{m}}})$ is achieved when ${\lambda }_{{\rm{LSPR}}}$ is approximately 904 nm, near the J-aggregate exciton resonance, which is approximately 4.1 times that obtained at ${\lambda}_{\mathrm{LSPR}}=806\,\mathrm{nm}$, near the monomer exciton resonance. The spectral responses of linear and nonlinear absorption mediated by the PRET of the hybrids can be well reproduced by our developed semiclassical theoretical model. Our theoretical analysis reveals that the largest PRET is attributed to the superposition of multiple plasmon–exciton Fano interferences associated with the strong saturable absorption (SA) of the J-aggregate excitons.

## Results

**Fig. 1:**
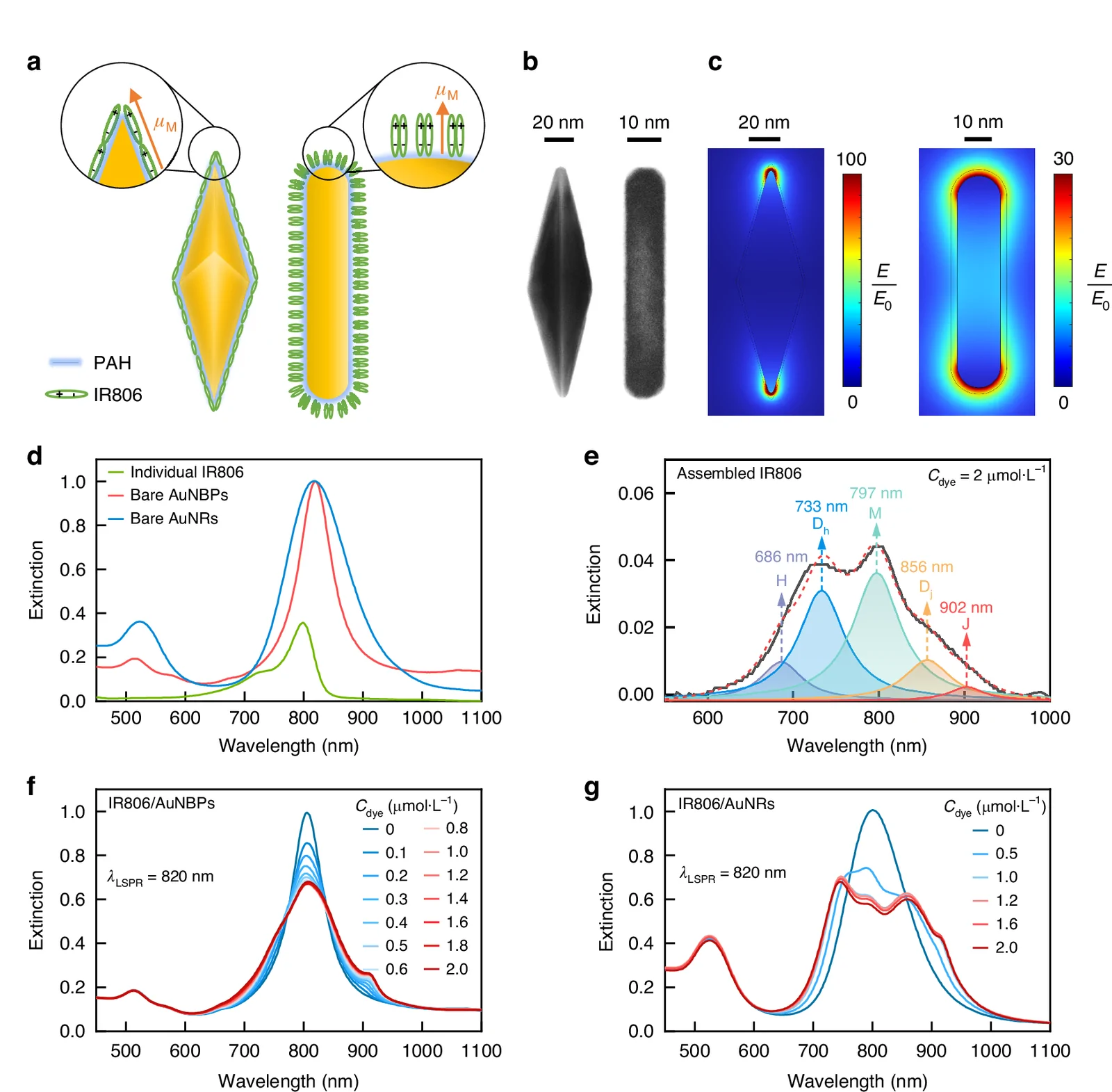
Nanostructure and multiple plasmon–exciton Fano interferences of the IR806/AuNBP and IR806/AuNR hybrids. **a** Schematic of IR806/AuNBP and IR806/AuNR hybrid nanostructures. **b** Transmission electron microscopy (TEM) images of single IR806/AuNBP (${\lambda }_{{\rm{LSPR}}}=904$ nm) and IR806/AuNR (${\lambda }_{{\rm{LSPR}}}=888$ nm). **c** Simulated electric distributions of single IR806/AuNBP and IR806/AuNR with ${\lambda }_{{\rm{LSPR}}}=900$ nm at the Fano dip (λ=900 nm). **d** Extinction spectra of bare AuNBPs and AuNRs with ${\lambda }_{{\rm{LSPR}}}=820$ nm, compared with those of individual IR806 molecules showing monomer exciton resonances. **e** Extinction spectra of assembled IR806 molecules with five exciton resonances (H, D_h_, M, D_j_, and J) in the presence of PAH. **f** Extinction spectra of the IR806/AuNBP as a function of the molecular concentration ${C}_{{\rm{dye}}}$. **g** IR806/AuNR hybrids (${\lambda }_{{\rm{LSPR}}}=820$ nm) vary with molecular concentration ${C}_{{\rm{dye}}}$. Prominent asymmetric Fano interference appears at approximately 910 nm for the IR806/AuNBP hybrids, whereas three distinct Fano dips are observed at 770, 820, and 900 nm for the IR806/AuNR hybrids

IR806 molecules are assembled on the surface of the AuNBPs and AuNRs using poly (allylamine hydrochloride) (PAH) as a molecular linker. The AuNBPs possess flat facets, whereas the AuNRs have smooth curved surfaces (Fig. S1). These distinct nanostructures result in different molecular orientations of IR806 molecules on their respective surfaces^38^. Specifically, the J-aggregate excitons of IR806 molecules align parallel to the surface of the AuNBPs, while the H-aggregate dimers (D_h_) and J-aggregate dimers (D_j_) excitons adopt a perpendicular configuration on the surface of the AuNRs, as illustrated in Fig. 1a, b. Differences in nanostructures strongly affect the electric field. In the AuNBPs, the electric field is strongly localized at the sharp tips, whereas in the AuNRs, it is concentrated mainly at the round ends. In both systems, energy is redistributed into the molecular layer through destructive Fano interference between plasmons and excitons. Owing to the more concentrated hot spot at the sharp tips, the local electric field of IR806/AuNBPs is approximately three times stronger than that of IR806/AuNRs for the equivalent LSPR wavelength of 900 nm (${\lambda }_{\mathrm{LSPR}}=900\,\mathrm{nm}$) (Fig. 1c). The hot spots within the molecular layers contribute predominantly to destructive Fano interference (Figs. S2–S4).

The IR806/AuNBPs and IR806/AuNRs display in-phase dipolar charge patterns at 890 and 930 nm (separated by a global π flip). At 900 nm, however, the bipyramid develops a relatively large phase gradient and strong shell localization (Figs. S2 and S3). In addition, although the bare AuNBPs and AuNRs have the same resonance wavelength and intensity (${\lambda }_{\mathrm{LSPR}}=820$ nm; σ=1), as shown in Fig. 1d, the full width at half maximum of the AuNBPs is smaller, resulting in a higher quality factor Q of the LSPR and stronger nonlinear absorption. The individual IR806 molecules in aqueous solution exhibit a primary absorption peak at 800 nm, which corresponds to monomer excitons (labeled M), along with a shoulder peak near 730 nm due to molecular vibrations (Fig. 1d). However, upon assembly with PAH, the spectrum changes substantially, showing five distinct excitonic peaks at 686, 733, 797, 856, and 902 nm, which are assigned to H-aggregates (H), H-aggregate dimers (D_h_), monomers (M), J-aggregate dimers (D_j_), and J-aggregates (J), respectively^39,40^ (Figs. 1e and S5). The quality factors *Q* of the five excitons are 11.8 (H), 11.3 (D_h_), 11.8 (M), 13.2 (D_j_), and 18.6 (J). Notably, the J-aggregate excitons achieve the lowest resonance energy and the highest quality factor, implying a superior plasmon coupling PRET efficiency. As the molecular concentration of IR806 increases from 0 to 2 ${\rm{\mu }}{\rm{mol}}\,{{\rm{L}}}^{-1}$ in the IR806/AuNBP hybrids (Fig. 1f), the extinction near 800 nm decreases from 1.0 to 0.68, whereas the hybrid peak near 910 nm increases from 0.16 to 0.27. These changes result from destructive and constructive Fano interference of the plasmons and excitons, respectively. The coupling between the J-aggregate exciton and plasmon gives rise to a Fano dip at 902 nm in the IR806/AuNBP hybrids. In contrast, the IR806/AuNR hybrids show a more complex spectral lineshape. The extinction near the plasmon resonance at ${\lambda }_{{\rm{LSPR}}}=820$ nm gradually decreases, with three distinct Fano dips clearly observed at 770, 820, and 900 nm and four hybrid peaks at 746 (dominant), 796, 860, and 914 nm, respectively (Fig. 1g). These observations indicate that the distinct spectral responses of the IR806/AuNBP and IR806/AuNR hybrids fundamentally originate from the synergistic interaction between molecular orientation and localized plasmonic electric distributions, governed by their nanostructure.

**Fig. 2:**
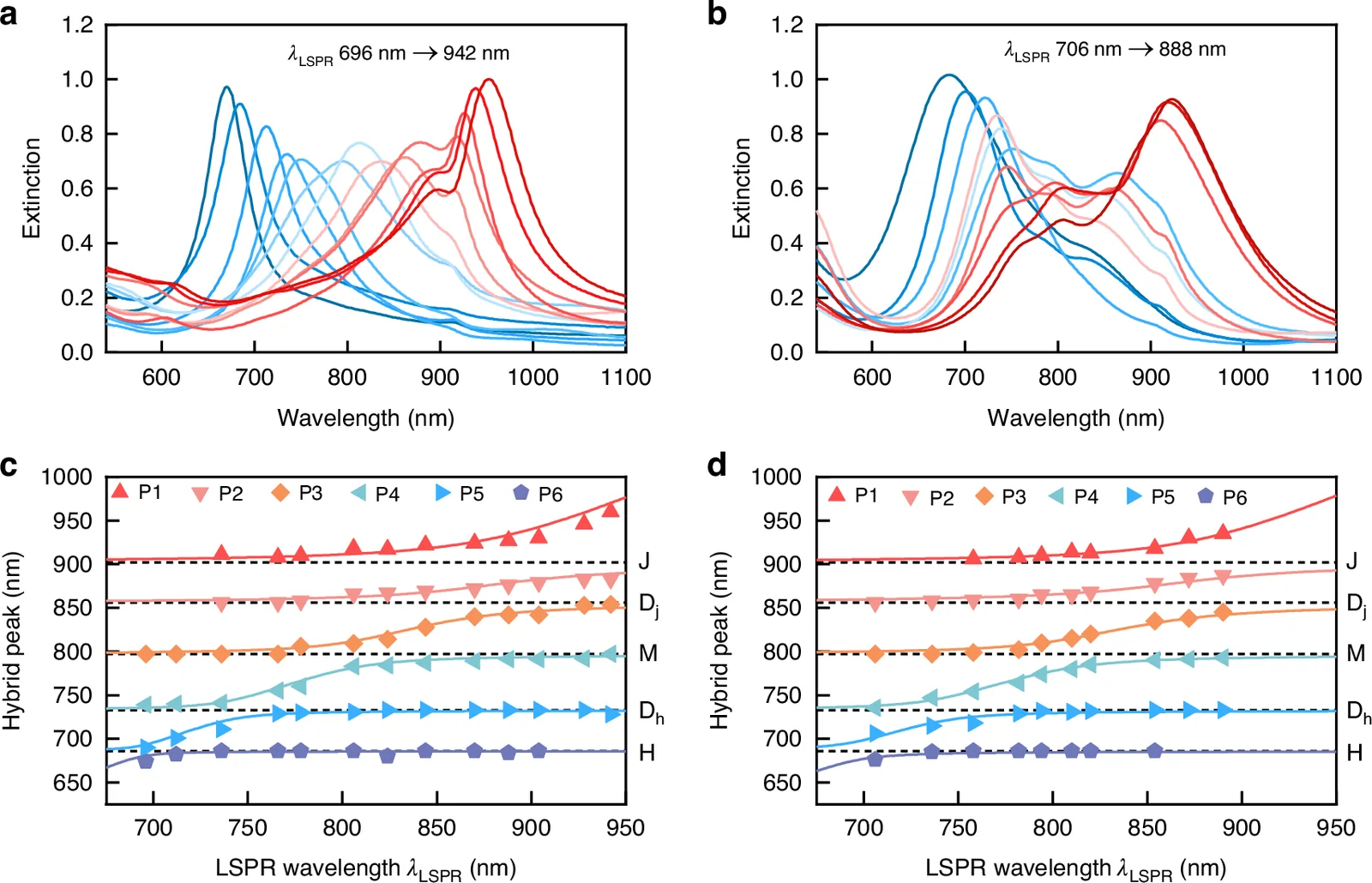
Multiple hybridized resonances of IR806/AuNBPs and IR806/AuNRs vary with the LSPR wavelength *λ*_LSPR_. **a** Extinction spectra of IR806/AuNBPs with ${\lambda }_{{\rm{LSPR}}}=$ 696, 712, 736, 766, 778, 806, 824, 844, 870, 888, 904, 928, and 942 nm. **b** Extinction spectra of IR806/AuNRs with *λ*_LSPR_ = 706, 736, 758, 782, 794, 810, 820, 872, and 888 nm. The extinction peak intensity of bare AuNBPs and AuNRs is fixed at 1.0, and the concentration of the IR806 molecules is fixed at $2\,{\rm{\mu }}\mathrm{mol}\,{{\rm{L}}}^{-1}$. **c** Six hybridized wavelengths of IR806/AuNBPs as a function of ${\lambda }_{{\rm{LSPR}}}$. The coupling constants of the plasmons and five excitons (H, D_h_, M, D_j_, and J) are calculated to be 17.6, 26.9, 33.3, 37.3, and 47.9 meV, respectively. **d** Six hybridized wavelengths of IR806/AuNRs as a function of ${\lambda }_{{\rm{LSPR}}}$. The coupling constants of the plasmons and five excitons (H, D_h_, M, D_j_, and J) are calculated to be 29.9, 33.1, 37.9, 45.5, and 43.5 meV, respectively

Additionally, differences are observed in the dye concentration-dependent extinction spectral evolution of IR806/AuNBPs and IR806/AuNRs at different ${\lambda }_{{\rm{LSPR}}}$ (Figs. S6 and S7). At a dye concentration of ${C }_{\mathrm{dye}}=2\,{\upmu}{\rm{mol}}\,{{\rm{L}}}^{-1}$, the extinction spectra of both hybrids exhibit six hybridized resonance peaks induced by multiple Fano interferences, as shown in Figs. S8 and S9. For the IR806/AuNBPs, the main resonance peak of the hybrids closely follows the LSPR wavelength ${\lambda }_{{\rm{LSPR}}}$ of the AuNBPs and redshifts from 670 to 954 nm as ${\lambda }_{{\rm{LSPR}}}$ increases from 696 to 942 nm. The main resonance intensity $\alpha ({\lambda }_{{\rm{hyb}}})$ of the hybrids also varies with the ${\lambda }_{{\rm{LSPR}}}$ of the AuNBPs. It remains near 1.0 when ${\lambda }_{{\rm{LSPR}}}$ is above 900 nm or below 700 nm, but considerably decreases for ${\lambda }_{{\rm{LSPR}}}$ between 730 and 870 nm (Fig. 2a). This ${\lambda }_{{\rm{LSPR}}}$-dependent behavior of $\alpha ({\lambda }_{{\rm{hyb}}})$ is primarily caused by the superposition of constructive and destructive Fano interference between the plasmons and five excitonic states, although variations in plasmon–exciton coupling strength also contribute. Similarly, the hybridized resonance peaks of IR806/AuNRs vary substantially with ${\lambda }_{{\rm{LSPR}}}$. Throughout the variation, three distinct Fano dips remain evident because of the multiple couplings between the plasmons and excitons (Fig. 2b). Moreover, the anticrossing relationships of six hybridized resonances with varying ${\lambda }_{{\rm{LSPR}}}$ are well reproduced by the eigenvalue equation of coupled oscillators with the Hamiltonian matrix (Fig. 2c, d).

1 $$H=\left(\begin{array}{llllll}{E}_{{\rm{p}}}^{{\prime} }-i{\gamma }_{{\rm{p}}}/2 & {\Delta }_{{\rm{H}}} & {\Delta }_{{{\rm{D}}}_{{\rm{h}}}} & {\Delta }_{{\rm{M}}} & {\Delta }_{{{\rm{D}}}_{{\rm{j}}}} & {\Delta }_{{\rm{J}}}\\ {\Delta }_{{\rm{H}}} & {E}_{{\rm{H}}}^{{\prime} }-i{\gamma }_{{\rm{H}}}/2 & 0 & 0 & 0 & 0\\ {\Delta }_{{{\rm{D}}}_{{\rm{h}}}} & 0 & {E}_{{{\rm{D}}}_{{\rm{h}}}}^{{\prime} }-i{\gamma }_{{{\rm{D}}}_{{\rm{h}}}}/2 & 0 & 0 & 0\\ {\Delta }_{{\rm{M}}} & 0 & 0 & {E}_{{\rm{M}}}^{{\prime} }-i{\gamma }_{{\rm{M}}}/2 & 0 & 0\\ {\Delta }_{{{\rm{D}}}_{{\rm{j}}}} & 0 & 0 & 0 & {E}_{{{\rm{D}}}_{{\rm{j}}}}^{{\prime} }-i{\gamma }_{{{\rm{D}}}_{{\rm{j}}}}/2 & 0\\ {\Delta }_{{\rm{J}}} & 0 & 0 & 0 & 0 & {E}_{{\rm{J}}}^{{\prime} }-i{\gamma }_{{\rm{J}}}/2\end{array}\right)$$

where ${E}_{{\rm{p}}}^{{\prime} }$, ${E}_{{\rm{H}}}^{{\prime} }$, ${E}_{{{\rm{D}}}_{{\rm{h}}}}^{{\prime} }$, ${E}_{{\rm{M}}}^{{\prime} }$, ${E}_{{{\rm{D}}}_{{\rm{j}}}}^{{\prime} }$, and ${E}_{{\rm{J}}}^{{\prime} }$ are the eigenvalues associated with the plasmons and five excitonic states corrected by the local dielectric environment, respectively. ${\gamma }_{{\rm{p}}}$, ${\gamma }_{{\rm{H}}}$, ${\gamma }_{{{\rm{D}}}_{{\rm{h}}}}$, ${\gamma }_{{\rm{M}}}$, ${\gamma }_{{{\rm{D}}}_{{\rm{j}}}}$, and ${\gamma }_{{\rm{J}}}$ represent the decay rates of plasmon and five excitonic states, respectively. Here, the broadening term γ can be neglected for simplification. ${\triangle }_{{\rm{H}}}$, ${\Delta }_{{{\rm{D}}}_{{\rm{h}}}}$, ${\triangle }_{{\rm{M}}}$, ${\Delta }_{{{\rm{D}}}_{{\rm{j}}}}$, and ${\triangle }_{{\rm{J}}}$ represent the corresponding coupling constants of the plasmons and excitons for IR806/AuNBPs, which are calculated to be 17.6, 26.9, 33.3, 37.3, and 47.9 meV, respectively. This coupled-oscillator Hamiltonian model can be used to describe both Fano interference in the intermediate coupling regime $\frac{1}{2}\left({\gamma }_{{\rm{p}}}-{\gamma }_{{\rm{c}}}\right) < 2\triangle \,\le \frac{1}{2}\left({\gamma }_{{\rm{p}}}+{\gamma }_{{\rm{c}}}\right)$ and Rabi splitting in the strong coupling regime $2\triangle \, > \,\frac{1}{2}\left({\gamma }_{{\rm{p}}}+{\gamma }_{{\rm{c}}}\right)$, where ${\gamma }_{{\rm{p}}}$ and ${\gamma }_{{\rm{c}}}$ are decay rates of plasmon and exciton, respectively^41,42^. A distinct Fano dip near 900 nm primarily arises from the coupling between plasmon and J-aggregate excitons. This coupling (47.9 meV), which is the greatest among the five observed, is attributed to the considerable longitudinal dipole moment component of the J-aggregate excitons. All five coupling constants remain smaller than the ${\gamma }_{{\rm{p}}}=153.2\,{\rm{meV}}$ at ${\lambda }_{{\rm{LSPR}}}=904$ nm, and these relatively small couplings of Fano interferences drive one-way energy transfer from plasmons to excitons (i.e., the PRET). In contrast, the D_h_ and D_j_ excitons play dominant roles in the IR806/AuNRs, with coupling constants of 33.1 meV and 45.5 meV, respectively.

The hybrids IR806/AuNBPs and IR806/AuNRs demonstrate different favorable coupling strengths owing to morphology-dependent exciton alignment. The coupling constant follows ${g}_{\rm{pc}}\propto {\upmu }_{\rm{c}}\cos \theta$, where θ is the angle between the exciton dipole and the local plasmonic field^3^. The sharp tips of AuNBPs preferentially stabilize J-aggregate excitons with the largest $\cos \theta$ for ${\triangle }_{{\rm{J}}}$, while the rounded ends of AuNRs favor D_h_/D_j_ excitons with the largest $\cos \theta$ for ${\Delta }_{{{\rm{D}}}_{{\rm{h}}}}$ and ${\Delta }_{{{\rm{D}}}_{{\rm{j}}}}$ (Fig. S10). This structural dependence highlights the critical role of molecular arrangement in governing plasmon–exciton interactions and resulting optical responses. Additionally, the ${\lambda }_{{\rm{LSPR}}}$ of the AuNBPs affects the intensity and wavelength of the exciton resonance generated by the IR806 molecules, which subsequently influences the coupling between the five exciton states and the plasmons (Fig. S11).

**Fig. 3:**
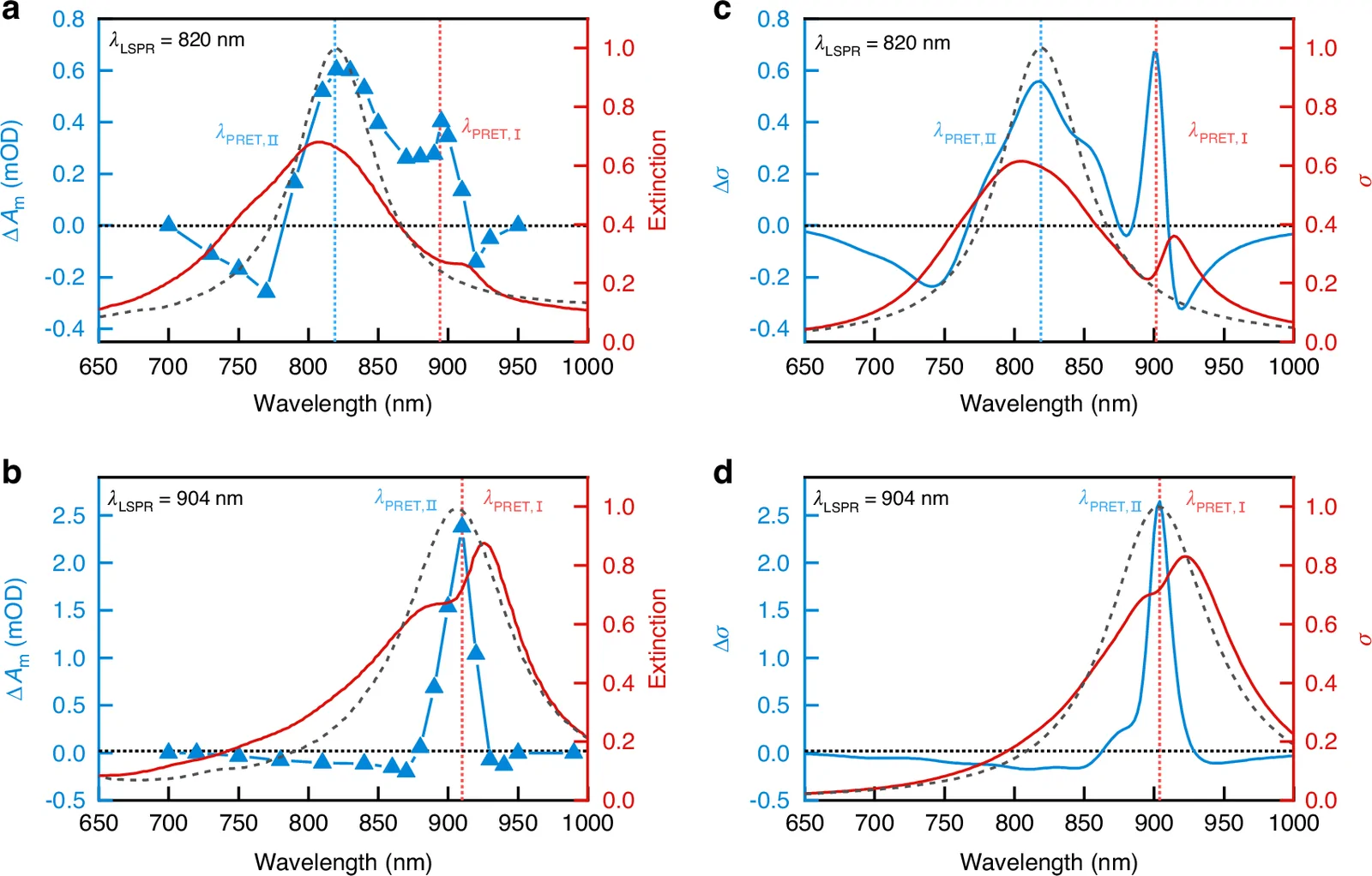
Linear and nonlinear absorption of the IR806/AuNBP hybrids. Measured differential absorption $\Delta {A}_{{\rm{m}}}(\lambda )$ (blue line) with extinction spectra of hybrids (red solid line) and bare nanostructures (gray dashed line) with ${\lambda }_{{\rm{LSPR}}}=820$ nm (**a**) and 904 nm (**b**). Calculated linear absorption $\sigma (\lambda )$ and nonlinear absorption $\Delta \sigma (\lambda )$ with ${\lambda }_{{\rm{LSPR}}}=820$ nm (**c**) and 904 nm (**d**)

To investigate the PRET in the IR806/AuNBP hybrids and the plasmon relaxation of the AuNBPs, ultrafast transient absorption spectroscopy measurements were performed. The transient differential transmittance $\triangle I(t)/{I}_{0}$ is measured as a function of delay t, and the transient differential absorbance ∆A(t) is then obtained from the equation $\triangle A(t)=-{\mathrm{lg}}[1+\triangle I(t)/{I}_{0}]$. The extremal value of ∆A(t) is denoted as $\Delta {A}_{{\rm{m}}}$. For the bare AuNBPs, $\Delta {A}_{{\rm{m}}}$ is negative around the LSPR wavelength owing to the SA of the bare plasmon (Fig. S12). The SA of bare IR806 molecules with PAH exhibits a very weak signal in pump-probe measurements (Fig. S13), and the saturated intensity of SA is measured by the Z-scan method and presented later. On the contrary, the IR806/AuNBP hybrids exhibit a positive $\Delta {A}_{{\rm{m}}}$, indicating that the RSA is driven by energy transfer from the plasmons to the excitons (i.e., the PRET). Additionally, the linear dependence of this RSA signal ∆A(t) on the excitation intensity in ultrafast pump-probe measurements indicates that the PRET observed in the hybrids is attributed to one-photon processes (not the two-photon processes)^21,43^ (Fig. S14). At ${\lambda }_{{\rm{LSPR}}}=820$ nm, the IR806/AuNBPs show RSA under excitation at$\,{\lambda }_{{\rm{exc}}}=820$ nm, whereas the bare AuNBPs display SA (Fig. S15a, b). The nonlinear absorption spectrum of IR806/AuNBPs (${\lambda }_{{\rm{LSPR}}}=820$ nm) reveals two RSA ($\Delta {A}_{{\rm{m}}} > 0$) peaks: one peak at *λ*_exc_ = 895 nm (${\lambda }_{{\rm{PRET}},{\rm{{\rm I}}}}$) with $\Delta {A}_{{\rm{m}}}\left(\lambda \right)=+0.4$ mOD and the other at *λ*_exc_ = 820 nm (*λ*_PRET,II_), with a stronger $\Delta {A}_{{\rm{m}}}(\lambda )$ value of +0.6 mOD (Fig. 3a). These features are attributed to destructive plasmon–exciton Fano interference near the J-aggregate resonance. Notably, the peak of nonlinear absorption $\Delta {A}_{{\rm{m}}}(\lambda )$ is much more prominent than the Fano dip of extinction $\alpha (\lambda )$. The value of ${\lambda }_{{\rm{PRET}},{\rm{II}}}$ is slightly larger than the extinction peak wavelength (${\lambda }_{{\rm{hyb}}}=806$ nm) of the hybrids, which originates from constructive Fano interference. Moreover, the energy transfer between the AuNBPs and IR806 molecules can be modulated by tuning the ${\lambda }_{{\rm{LSPR}}}$. The results of transient differential absorption spectroscopy of IR806/AuNBPs and bare AuNBPs (${\lambda }_{{\rm{LSPR}}}=904$ nm) under different excitation wavelengths are shown in Fig. S15c and d. When ${\lambda }_{{\rm{LSPR}}}=904$ nm, the overlap of the two PRET peaks leads to a single RSA peak of nonlinear absorption, which corresponds to the Fano dip in the linear absorption spectrum (Fig. 3b). The overlapped PRET with an enhanced $\Delta {A}_{{\rm{m}}}(\lambda )$ of $+2.38\,\mathrm{mOD}$ is primarily attributed to the superposition of multiple Fano interferences between the plasmons and excitons, as well as the SA of the J-aggregates near 900 nm.

The power-dependent transmittance and effective two-photon absorption coefficient ${\beta }_{{\rm{eff}}}$ of the bare IR806 molecules, bare AuNBPs, and IR806/AuNBP hybrids are comparatively explored by open-aperture Z-scan measurements^44,45^ at the J-exciton resonance wavelength of 904 nm. For the 2 ${\rm{\mu }}\mathrm{mol}\,{{\rm{L}}}^{-1}$ bare IR806 molecules with PAH, we obtained the nonlinear coefficient of ${\beta }_{\mathrm{eff},i={\rm{J}}}=-0.727\,\mathrm{cm}\,{\mathrm{GW}}^{-1}$, and the corresponding saturated intensity of ${I}_{{\rm{S}},i={\rm{J}}}\approx -\frac{{{\rm{\alpha }}}_{0,i={\rm{J}}}}{{\beta }_{\mathrm{eff},i={\rm{J}}}}=0.058\,\mathrm{GW}\,{\mathrm{cm}}^{-2}$. For the bare AuNBPs (${\lambda }_{{\rm{LSPR}}}=904$ nm), ${\beta }_{{\rm{eff}},{\rm{p}}}$ is $-0.95\,\mathrm{cm}\,{\mathrm{GW}}^{-1}$, and the saturated intensity of ${I}_{{\rm{S}},{\rm{p}}}\approx -\frac{{{\rm{\alpha }}}_{0,{\rm{p}}}}{{\beta }_{{\rm{eff}},{\rm{p}}}}$ is about $2.43\,\mathrm{GW}\,{\mathrm{cm}}^{-2}$. The saturated intensity of bare IR806 molecules is approximately two orders of magnitude smaller than that of the bare AuNBPs. For the IR806/AuNBP hybrids, ${\beta }_{{\rm{eff}}}$ equals $+2.55\,\mathrm{cm}\,{\mathrm{GW}}^{-1}$, owing to RSA induced by PRET from plasmons to excitons (Fig. S16).

Furthermore, to elucidate the physical mechanism of the PRET driven by multiple plasmon–exciton Fano interferences, we calculate the absorption spectra and the corresponding nonlinear response of the hybrid system using a newly developed semiclassical model. This model builds upon previously established linear response theory^27,46^, and the linear absorption with neglect of coupling between molecules and the external field is equivalent to the method introduced by Manjavacas et al.^47^. The total power-dependent absorption of the hybrid system, denoted as σ(I), can be decomposed into three terms, $\sigma ={\sigma }_{{\rm{pp}}}+{\sigma }_{{\rm{cc}}}+{\sigma }_{{\rm{pc}}}$, where ${\sigma }_{{\rm{pp}}}$ and ${\sigma }_{{\rm{cc}}}$ represent the modified absorptions of plasmon and molecules, respectively, while ${\sigma }_{{\rm{pc}}}$ describes intercrossing in the hybrids^46^. The plasmonic term ${\sigma }_{{\rm{pp}}}$ is generally dominant and is expressed as

2 $${\sigma }_{\mathrm{pp}}\left(I\right)\propto \frac{{n}_{{\rm{p}}}\left(I\right){V}_{{\rm{p}}}^{2}}{\left(\omega -{\omega }_{{\rm{p}}}+i{\gamma }_{{\rm{p}}}\right)-{\sum }_{i=1}^{5}\frac{{n}_{{\rm{p}}}\left(I\right){n}_{i}\left(I\right){V}_{i}^{2}}{\left(\omega -{\omega }_{i}+i{\gamma }_{i}\right)}}$$

where ${\omega }_{{\rm{p}}}$ and ${\gamma }_{{\rm{p}}}$ represent the resonance frequency and linewidth of the plasmon, respectively; ${\omega }_{i}\,\left(i=\mathrm{1,2,3,4,5}\right)$ and ${\gamma }_{i}$ correspond to the resonance frequency and linewidth of the *i-*th exciton; ${V}_{{\rm{p}}}$ represents the interaction between the plasmon and the external field; ${V}_{i}$ describes the interaction between the plasmon and the *i-*th exciton; and ${n}_{{\rm{p}}}$ and ${n}_{i}$ characterize the number of plasmons and excitons involved in the interaction, respectively. These parameters change with increasing excitation power I, resulting in nonlinear absorption. We assume that their population dynamics follow the SA effect, given by ${n}_{{\rm{p}}}={n}_{{\rm{p}}0}/(1+{I/I}_{{\rm{S}},{\rm{p}}})$ and ${n}_{i}={n}_{i0}/(1+{I/I}_{{\rm{S}},i})$, where ${I}_{{\rm{S}},{\rm{p}}}$ and ${I}_{{\rm{S}},i}$ are the saturation intensity of the plasmons and excitons, respectively. This saturation assumption implies that plasmonic and excitonic modes remain well-defined elementary excitations, and therefore applies to the non-strong-coupling regime. The saturation power depends on frequency ω, linear absorption ${\sigma }_{0}$, and lifetime τ according to the relationship ${I}_{{\rm{S}}}\propto \hslash \omega /{\sigma }_{0}\tau$. In the coupled system, the effective molecular saturation intensities ${I}_{{\rm{S}},i}$ are taken to follow the scaling relation above and are treated as configuration dependent due to plasmon-enhanced local fields, while the plasmonic channel is considered effectively unsaturated in the full-spectrum simulations (see Supplementary Note 6). Then, the differential absorption spectrum is calculated by

3 $$\Delta \sigma \left(I\right)=\sigma \left(I\right)-\sigma \left(I=0\right)$$

The calculated linear and nonlinear absorption spectra ($\sigma \left(\lambda \right)$ and $\triangle \sigma \left(\lambda \right)$) of the hybrids with ${\lambda }_{{\rm{LSPR}}}=820$ nm are shown in Fig. 3c. Our calculations effectively reproduce the experimental observations of three prominent extinction peaks and two RSA peaks (Fig. 3a). A weak extinction peak is observed at 915 nm, accompanied by a PRET peak I at ${\lambda }_{{\rm{PRET}},{\rm{{\rm I}}}}=904$ nm, which is attributed mainly to the constructive and destructive Fano interference between the plasmons and J-aggregate excitons. The main hybrid extinction peak appears at ${\lambda }_{{\rm{hyb}}}=805$ nm, while PRET peak II is located at *λ*_PRET,II_ = 820 nm. This PRET peak II wavelength is close to the ${\lambda }_{{\rm{LSPR}}}$ of the AuNBPs, indicating efficient plasmon–exciton energy transfer. For IR806/AuNBPs with ${\lambda }_{{\rm{LSPR}}}=904$ nm, the theoretical linear absorption exhibits a distinct Fano dip at the J-aggregate exciton resonance, which is consistent with the experimental observations in Fig. 3b. The semiclassical model fits the experiment well, revealing that the RSA peak originates from the overlap of the two PRET peaks (Fig. 3d).

**Fig. 4:**
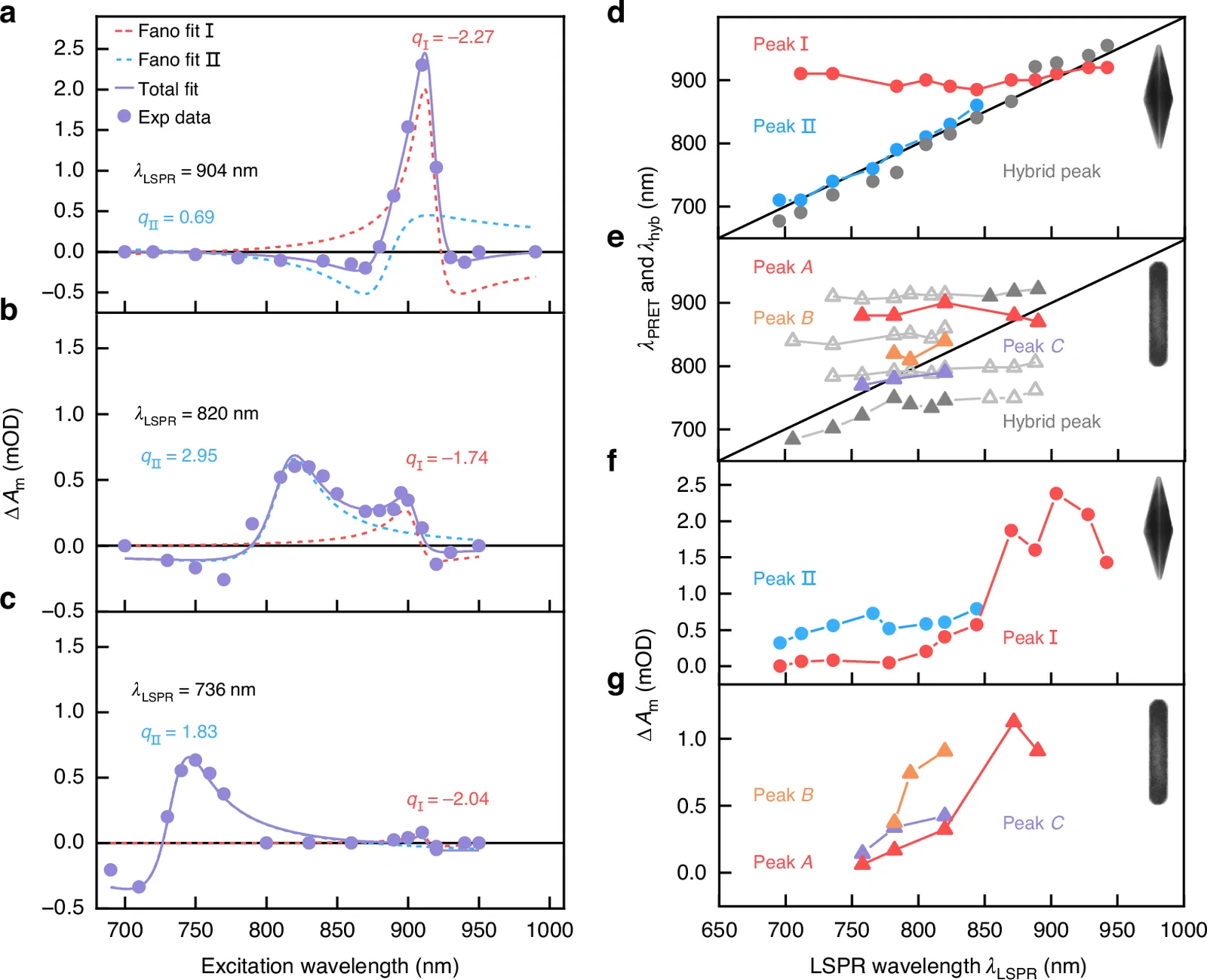
Fano lineshape fitting of the nonlinear absorption spectra of IR806/AuNBPs, and the nonlinear resonance wavelength and PRET peak intensity of the IR806/AuNBPs and IR806/AuNRs as a function of the *λ*_LSPR_. **a**–**c** Measured differential absorption spectra $\triangle {A}_{{\rm{m}}}(\lambda )$ and corresponding Fano lineshape fitting of the IR806/AuNBPs with ${\lambda }_{{\rm{LSPR}}}=$ 904, 820, and 736 nm, respectively. Measured peak wavelengths ${\lambda }_{{\rm{PRET}}}$ of nonlinear absorptions accompanied by peak wavelengths ${\lambda }_{{\rm{hyb}}}$ of linear absorptions of IR806/AuNBPs (**d**) and IR806/AuNRs (**e**) as a function of ${\lambda }_{{\rm{LSPR}}}$. Differential absorption intensities $\triangle {A}_{{\rm{m}}}$ at ${\lambda }_{{\rm{PRET}}}$ of IR806/AuNBPs (**f**) and IR806/AuNRs (**g**) as a function of ${\lambda }_{{\rm{LSPR}}}$

Although the concept of a Fano lineshape originally refers to linear absorption, the nonlinear absorption spectra here display inverted Fano-like features. This apparent inversion occurs because SA suppresses strong resonances in the linear spectra; therefore, the nonlinear differential response inherits the Fano-type characteristics but with opposite signs at the resonance positions where absorption is strongest. These nonlinear spectra can be well fitted by two Fano profiles with q parameters of opposite signs, where the fitted Fano peak I corresponds to the PRET peak I and the fitted Fano peak II aligns with the PRET peak II (Fig. 4a–c). When the plasmonic resonance is tuned across the molecular resonances, the phase character of the plasmon-induced polarization reverses, thereby flipping the sequence of constructive and destructive interference for different exciton modes (q=0 when the detuning between plasmon and exciton resonances is zero). The Fano parameter $q=\cot \delta$ depends on the phase shift$\,\delta$ between the plasmon and exciton^48^, which governs the Fano resonance interaction and shapes the distinct spectral profiles. The Fano lineshape profile I primarily describes plasmon coupling with the J-aggregate exciton and shows a negative Fano parameter (${q}_{{\rm{{\rm I}}}} < 0$) corresponding to a phase shift exceeding ${\rm{\pi }}/2$. In contrast, the Fano lineshape profile II simulates the nonlinear absorption of plasmon coupling with multiple excitons, resulting in a positive Fano parameter (*q*_II_ > 0). This result indicates a phase shift smaller than π/2. Additional nonlinear absorption spectra of IR806/AuNBPs with different *λ*_LSPR_ are also provided in Fig. S17.

A comparison of the nonlinear absorption spectra of the IR806/AuNBP hybrids with different values of ${\lambda }_{{\rm{LSPR}}}$ reveals that the PRET peak II wavelength is bound around ${\lambda }_{{\rm{LSPR}}}$ of the AuNBPs, which can be tuned from 710 to 860 nm. However, the PRET peak I remains nearly fixed around the J-aggregate resonance at 910 nm. The main PRET wavelength ${\lambda }_{{\rm{PRET}}}$ associated with destructive Fano interference is slightly redshifted relative to ${\lambda }_{{\rm{hyb}}}$ when ${690 < \lambda }_{\mathrm{LSPR}} < 880\,\mathrm{nm}$. However, ${\lambda }_{{\rm{PRET}}}$ becomes slightly smaller than ${\lambda }_{{\rm{hyb}}}$ of constructive interference for the IR806/AuNBPs with ${\lambda }_{{\rm{LSPR}}}$ around the J-aggregate exciton resonance (${\lambda }_{{\rm{LSPR}}} > 880$ nm) (Fig. 4d). This variation in the relative positions of ${\lambda }_{{\rm{PRET}}}$ and ${\lambda }_{{\rm{hyb}}}$ is caused by the complete effects of the superposition of multiple Fano interferences of the plasmons with five excitonic states in the hybrids. The transient absorption spectra of IR806/AuNBPs with different values of ${\lambda }_{{\rm{LSPR}}}$ at the maximum RSA effect (${\lambda }_{{\rm{PRET}}}$) are provided in Fig. S18. In the nonlinear absorption spectrum of the IR806/AuNRs, three PRET peaks appear at approximately 900, 820, and 770 nm, denoted as PRET peaks *A*, *B*, and *C*, respectively (Fig. 4e). These values correspond to the Fano dips in the linear absorption spectrum. The detailed transient differential absorption spectra of the IR806/AuNR hybrids are presented in Fig. S19.

Most importantly, the peak of nonlinear absorption $\triangle {A}_{{\rm{m}}}\,(\lambda ={\lambda }_{{\rm{PRET}}})$ induced by the PRET is highly tunable by adjusting the ${\lambda }_{{\rm{LSPR}}}$ of bare AuNBPs (Fig. 4f). When ${\lambda }_{{\rm{LSPR}}} < 850$ nm, the intensity of PRET peak II fluctuates near 0.5 mOD, whereas that of PRET peak I gradually increases, approaching the intensity of peak II. At ${\lambda }_{{\rm{LSPR}}}=904$ nm, the PRET peak reaches its maximum $\triangle {{{A}}}_{{\rm{m}}}$ because of the superposition of multiple Fano interferences between the plasmons and excitons. Surprisingly, this enhancement is dominated mainly by plasmon and J-aggregate exciton coupling at ${\lambda }_{{\rm{PRET}}}=910$ nm for the IR806/AuNBPs. The IR806/AuNRs exhibit maximum RSA intensity at ${\lambda }_{{\rm{LSPR}}}=872$ nm. This enhancement results from the superposition of multiple Fano interferences, predominantly induced by the D_j_ exciton. This understanding is supported by the strongest coupling constant of 45.5 meV between the Dⱼ exciton and the plasmon (Fig. 4g).

## Conclusions

In summary, we developed IR806/AuNBP and IR806/AuNR hybrids to investigate plasmon–exciton coupling. We find that the hybridization between the longitudinal LSPR of the nanoparticles and the five excitonic states of the assembled IR806 (H, Dₕ, M, Dⱼ, and J) gives rise to multiple Fano interferences. Moreover, distinct linear and nonlinear absorption properties are also observed, arising from differences in the orientation and aggregation of IR806 molecules on the AuNR and AuNBP surfaces. In the IR806/AuNBP hybrids, plasmon–exciton coupling is dominated by J-aggregates, whereas in the IR806/AuNR hybrids, the D_j_ and D_h_ excitons play the primary role. Additionally, the PRET can be tuned by adjusting the plasmon resonance wavelength ${\lambda }_{{\rm{LSPR}}}$ of the AuNBPs and AuNRs. The total PRET reaches a maximum at ${\lambda }_{{\rm{LSPR}}}=904$ nm, which is caused mainly by the coupling between plasmons and J-aggregate excitons with a high-quality factor. Compared with IR806/AuNRs, IR806/AuNBPs have a higher PRET maximum, where a PRET maximum is similarly detected near the D_j_ exciton resonance but with a smaller magnitude. Our theoretical calculations further confirm the superior tunability and high efficiency of IR806/AuNBPs. These findings offer considerable potential for advanced applications in ultrafast optical information processing and highly sensitive photodetectors.

## Materials and methods

### Materials

Gold chloride trihydrate (HAuCl_4_∙H_2_O, 99.9%), sodium borohydride (NaBH_4_, 98%), sodium citrate dihydrate (C_6_H_5_Na_3_O_7_·2H_2_O, 99%), hexadecyl trimethyl ammonium bromide (CTAB, 99%), cetyl trimethyl ammonium chloride (CTAC, 99.5%), hydrogen peroxide solution (H_2_O_2_, 30 wt%), and 2-[2-[2-chloro-3-[2-[1,3-dihydro-3,3-dimethyl-1-(4-sulfobutyl)-2H-indol-2-ylidene]-ethylidene]-1-cyclopenten-1-yl]-ethenyl]-3,3-dimethyl-1-(4-sulfobutyl)-3H-indolium hydroxide (inner salt sodium salt, IR806 dye content 90%) were purchased from Shanghai Aladdin Biochemical Technology Co., Ltd. Silver nitrate (AgNO_3_, 99.8%) and hydrochloric acid (HCl, 36–38%) were obtained from Yonghua Chemical Co., Ltd. Ascorbic acid (AA, 99%) was obtained from TCI (Shanghai) Development Co., Ltd. Sodium chloride (NaCl, 99.5%) was purchased from Shanghai Macklin Biochemical Co., Ltd. Poly (allylamine hydrochloride) (PAH, Mw approximately 50,000 g mol^−1^) was obtained from Sigma-Aldrich. Ultrapure water with a resistivity of approximately 18.2 MΩ cm was used as the solvent in all the experiments.

### Synthesis of AuNBPs

AuNBPs with various aspect ratios were synthesized using the seed-mediated growth method^49^. First, a citrate-stabilized seed solution was prepared by sequentially adding HAuCl_4_ (0.01 mol L^−1^, 0.125 mL), trisodium citrate (0.01 mol L^−1^, 0.25 mL), and a freshly prepared ice-cold NaBH_4_ solution (0.01 mol L^−1^, 0.15 mL) into ultrapure water (9.625 mL) under vigorous stirring. The mixture was stirred for 10 min and kept in a constant-temperature chamber at 35 °C for 2 h. The growth solution was prepared by sequentially adding HAuCl_4_ (0.01 mol L^−1^, 2 mL), AgNO_3_ (0.01 mol L^−1^, 0.4 mL), HCl (1 mol L^−1^, 0.8 mL), and AA (0.1 mol L^−1^, 0.32 mL) to 40 mL of 0.1 mol L^−1^ CTAB solution. The seed solution (0.1–2 mL) was then rapidly injected into the growth solution, followed by gentle inversion mixing for 10 s. The mixture was left undisturbed in a constant-temperature chamber at 35 °C for 8–12 h to obtain the AuNBPs. The as-grown AuNBPs were centrifuged at 6000–8000 rpm for 10 min. The precipitate was redispersed in a CTAC solution (0.08 mol L^−1^, 30 mL). AgNO_3_ (0.01 mol L^−1^, 6 mL) and ascorbic acid (0.1 mol L^−1^, 3 mL) were subsequently added to the solution, and the mixture was kept in an oven at 65 °C for 4 h to form Au/Ag bimetallic products. The bimetallic products were centrifuged at 5000 rpm for 10 min and redispersed in CTAB solution (0.05 mol L^−1^, 30 mL). The solution was left to stand in a constant-temperature chamber at 35 °C for 4 h. The supernatant was discarded, and the remaining Au/Ag bimetallic products were redispersed in 20 mL of water. Final purification was achieved by sequential addition of NH_3_·H_2_O (30 wt%, 4 mL) and H_2_O_2_ (0.1 mol L^−1^, 3 mL), followed by centrifugation at 6000–8000 rpm of clear supernatant for 10 min. The purified products were stored in a CTAB solution (0.05 mol L^−1^, 10 mL) or water for further use.

### Synthesis of AuNRs

The seed solution for AuNRs was also synthesized using the seed-mediated growth method^50^. Under continuous stirring, CTAB (0.2 mol L^−1^, 5 mL), HAuCl_4_ (5 mmol L^−1^, 500 ${\rm{\mu }}{\rm{L}}$), and freshly prepared ice-cold NaBH_4_ solution (0.01 mol L^−1^, 610 ${\upmu}{\rm{L}}$) were sequentially added to 4.5 mL of water. After 10 min, a brownish-yellow seed solution formed, which was stored at 35 °C for 2 h. A growth solution was prepared by sequentially adding the following components to the CTAB solution (0.2 mol L^−1^, 6 mL): HAuCl_4_ (5 mmol L^−1^, 1.2 mL), AgNO_3_ (0.1 mol L^−1^, 12 ${\rm{\mu }}{\rm{L}}$), HCl (1.2 mol L^−1^, 7 ${\rm{\mu }}{\rm{L}}$), and AA (0.01 mol L^−1^, 700 ${\rm{\mu }}{\rm{L}}$). The seed solution (8 ${\rm{\mu }}{\rm{L}}$) was subsequently rapidly injected into the growth solution, followed by gentle inversion mixing for 10 s. The resulting mixture was then placed in a constant-temperature incubator and stored for 8 h to form AuNRs. The resulting product was centrifuged at 10,000 rpm for 10 min, and the precipitate was redispersed in 6 mL of water. The size of the AuNRs can be adjusted by varying the amount of AgNO_3_ added.

### Preparation of IR806/AuNBPs and IR806/AuNRs

PAH (0.01 g) was dissolved in 1.0 mL of 1 mmol L^−1^ NaCl. This solution, along with 0.5 mL of 10 mmol L^−1^ NaCl, was added to 5 mL of an aqueous solution of AuNBPs (AuNRs), followed by gentle stirring for 1 h. The solution was then centrifuged (10,000 rpm, 10 min) and dispersed in 5 mL of ultrapure water to obtain PAH@AuNBPs (PAH@AuNRs). One milliliter of PAH@AuNBPs (PAH@AuNRs) was added to 1 mL of an IR806 aqueous solution at a specific concentration. The mixture was left to stand at room temperature for 30 min, after which the AuNBPs (AuNRs) were coupled with the near-infrared dye IR806. The resulting IR806/AuNBPs (IR806/AuNRs) were directly used for subsequent experiments without further centrifugation or dilution.

### Characterization

The morphologies of the IR806/AuNR and IR806/AuNBP samples were characterized using transmission electron microscopy (TEM; Hitachi HT7700) at an accelerating voltage of 100 keV. The extinction spectra were measured with a UV‒Vis‒NIR spectrophotometer (Persee T600B) using a tungsten lamp and a deuterium lamp as light sources, with a spectral bandwidth of 2 nm. The ultrafast dynamics of IR806/AuNBPs were investigated using a custom-built monochromatic transient absorption spectroscopy system. A Ti:sapphire laser (wavelength range: 680–1080 nm; repetition rate: 80 MHz; pulse width: 140 fs) was employed as the fundamental light source. The fundamental beam was split into two beams (pump and probe) via a beam splitter cube. The pump and probe beams were focused onto the sample solution using lenses with focal lengths of 100 and 150 mm, respectively, ensuring spatial overlap at the sample position. A delay line controlled by a stepper motor was used to adjust the time delay of the probe beam relative to the pump beam. The intensity of the probe beam after passing through the sample was recorded by a photodetector. A chopper (900 Hz) was used to modulate the pump beam, and a lock-in amplifier was employed to extract the weak transient absorption signals of the probe beam, thereby improving the signal-to-noise ratio. In all the experiments, the pump fluence was consistently maintained at 9.3 μJ cm^−2^ with a pump-to-probe energy fluence ratio of 25.8:1.

## Supplementary information

Supplementary Information for Tuning superposition of multiple Fano interferences for efficient plasmon resonance energy transfer to the assembled molecules

Dataset 1

## Data Availability

The authors declare that the data supporting the findings of this study are available within the article and its Supplementary Information files.
