# Supplementary material for: Tuning superposition of multiple Fano interferences for efficient plasmon resonance energy transfer to the assembled molecules
Source: Light Sci Appl. 2026 Sep 9;15:376. doi: 10.1038/s41377-026-02381-8 (PMC13558751; doi:10.1038/s41377-026-02381-8)
Supplement: Supplementary file 1 [file 41377_2026_2381_MOESM1_ESM.docx]

**Supplementary Information for**

**Tuning superposition of multiple Fano interferences for efficient plasmon resonance energy transfer to the assembled molecules**

Yishuang Wang^1†^, Xueqing Sang^1†^, Zhen-Long Dou^1,2†^, Qin-Xing Zhou^1^, Zhiyu Zhao^1^, Da-Jie Yang^3^, Yan Zhang^4^, Li Zhou^2*^, Xiaoguang Li^5*^, Qu-Quan Wang^1,4,6*^

^1^Department of Physics, State Key Laboratory of Quantum Functional Materials, and Guangdong Basic Research Center of Excellence for Quantum Science, Southern University of Science and Technology, Shenzhen 518055, China

^2^Key Laboratory of Artificial Micro- and Nano-Structures of the Ministry of Education, Hubei Nuclear Solid Physics Key Laboratory, School of Physics and Technology, Wuhan University, Wuhan 430072, China

^3^School of Mathematics and Physics, North China Electric Power University, Beijing 102206, China

^4^Quantum Science Center of Guangdong–Hong Kong–Macao Greater Bay Area (Guangdong), Shenzhen 518045, China

^5^Institute for Advanced Study, Shenzhen University, Shenzhen 518060, China

^6^Guangdong Provincial Key Laboratory of Advanced Thermoelectric Materials and Device Physics, Southern University of Science and Technology, Shenzhen 518055, China

^†^These authors contributed equally to this work

^*^Contact author: [qqwang@sustech.edu.cn](mailto:qqwang@sustech.edu.cn), xgli@szu.edu.cn, zhouli@whu.edu.cn

# This File Includes

Supplementary Notes 1 to 7

1. Morphologies of IR806/AuNRs and IR806/AuNBPs

2. Numerical Calculation of IR806/AuNBPs and IR806/AuNRs

3. Spectral Characterization and Resonance Hybridization

4.	Tuning Multiple Fano Interferences via Molecular Configuration and Orientation Control

5. Ultrafast Transient Absorption and Z-Scan Nonlinear Transmittance Measurements

6. Theory and Calculation of Linear and Nonlinear Absorption Spectra

7. References

# Supplementary Note 1. Morphologies of IR806/AuNRs and IR806/AuNBPs

The AuNBPs are structured as a combination of two congruent regular pentagonal pyramids. The average height is 157.3 nm, and the average radius is 24.5 nm. The AuNRs are rod-shaped nanostructures composed of a cylindrical body with two hemispherical end caps, with an average height of 67.0 nm, an average diameter of 14.4 nm, and a cap length of 5.6 nm. The IR806 molecular layer coated on the surfaces of the AuNBPs and AuNRs has a thickness of approximately 2 nm.

**Fig. S1.** **a** Transmission electron microscopy (TEM) image of IR806/AuNBPs with $\lambda_{\mathrm{LSPR}}=\text{90}\text{4}\text{ nm}$. **b** Structural schematic of AuNBPs with $\lambda_{\mathrm{LSPR}}=\text{90}\text{4}\text{ nm}$. **c** TEM image of IR806/AuNRs with $\lambda_{\mathrm{LSPR}}=\text{888 nm}$. **d** Structural schematic of AuNRs with $\lambda_{\mathrm{LSPR}}=\text{888 nm}$.

# Supplementary Note 2. Numerical Simulation of IR806/AuNBPs and IR806/AuNRs

## 2.1 Simulation Method

All the simulations are performed using the frequency domain module of COMSOL Multiphysics (v6.0). The complex permittivity of Au is taken from the experimental data of Johnson and Christy^1^ and interpolated onto a cubic spline in the range of 700–1100 nm. The background medium is water ($n_{\mathrm{bg}}=\text{1.33}$), and each particle is conformally coated with a 2 nm-thick molecular dye layer. The dielectric function of this dye layer is represented by a single Drude-Lorentz oscillator. The molecular polarizability is written as the sum of a constant part^2^, which describes the electronic polarization at high frequency, and a part caused by the molecular energy level transition:

$$\begin{aligned} \alpha\left( \omega\right)=\alpha_{\infty}+\alpha_{\text{t}}=\alpha_{\text{ e}}+\frac{\beta_{\text{t}}}{1-\omega^{2}/\omega_{0}^{2}-i\gamma\omega/\omega_{0}^{2}}\#\text{(S1)} \end{aligned}$$

where the transition component $\beta_{\text{t}}$ is given by

$$\begin{aligned} \beta_{\text{t}}=q^{2}/\epsilon_{0}\omega_{0}^{2}m\#\text{(S2)} \end{aligned}$$

For the investigated molecules, the effective charge is $q=\text{0.56}e$, and the reduced atom mass is $m=\text{2×}\text{10}^{\text{-26}}\mathrm{kg}$. To clarify the different optical responses of the hybridizations at approximately 900 nm, we only consider the transition $\hbar\omega_{0}\simeq\text{1.38}\mathrm{eV}$(900 nm). The polarizability of the whole molecular medium is defined as $\chi=N\alpha$, where *N* is the density of the molecules. The dielectric function is then expressed as^3^

$$\begin{aligned} \epsilon_{\text{dye}}=\epsilon_{\text{bg}}+\frac{N\alpha}{1-N\alpha/3}\approx\epsilon_{\text{bg}}+N{\alpha_{\text{e}}}_{\text{ }}+\frac{N\beta_{\text{t}}}{1-\omega^{2}/\omega_{0}^{2}-i\gamma\omega/\omega_{0}^{2}}\#\text{(S3)} \end{aligned}$$

where $\gamma=\text{0.069 eV}$ and $N=\text{0.067}$ nm^-3^ (2.5 times the molecular number density under standard conditions), which well reproduce the experimental results. Perfectly matched layers (PML) are applied at the outermost simulation boundary.

The incident plane wave is polarized parallel to the longitudinal axis of the AuNBPs or AuNRs, guaranteeing maximal excitation of the bright longitudinal dipole mode. To capture the steep field gradients within the 2 nm dye coating, a mesh with a target element size of 0.2 nm is employed throughout the dye shell. In the remaining domain, the element size is gradually increased to $\text{3 nm}$, preserving a growth factor $<\text{1.4}$.

## 2.2 Field and Charge Distributions of IR806/AuNBPs

The AuNBPs shown in Fig. S2a and b have a total tip-to-tip length of $L_{\mathrm{AuNBP}}=\text{158 nm}$ and an equatorial radius of $R_{\mathrm{AuNBP}}=\text{24.7 nm}$. The bare AuNBPs exhibit a longitudinal dipole resonance that spectrally overlaps with the absorption of the bare dye at approximately 900 nm. Upon coating the AuNBPs with the molecular shell, destructive interference between the bright plasmon mode and the dark excitonic transition gives rise to a Fano dip in the extinction spectrum (Fig. S2c). Snapshots of the electric field (Fig. S2d) and charge (Fig. S2e) reveal the underlying mechanism. Away from the minimum (890 nm and 930 nm), the hybrid supports a conventional dipolar mode: charges in the metal and dye oscillate synchronously, and the entire pattern simply undergoes a global $\pi$ phase flip between the two wavelengths. At the Fano dip (900 nm), however, a transitional state emerges: the charges in the metal core and the dye shell are no longer synchronous, and a phase gradient develops. This interlayer phase slip suppresses the radiated field (dip in extinction) while concentrating the energy in the shell, thereby enhancing the local field amplitude.

**Fig. S2.** **Fano resonance of AuNBPs coated with a 2-nm dye layer.** Geometric **a** and size **b** parameters of the AuNBPs. **c** Extinction cross section of the bare AuNBPs (red), the hybrid Au@dye structure (gray), and the isolated dye layer multiplied by $100$ for clarity (blue). Spectral overlap between the longitudinal dipole plasmon and the molecular resonance produces a characteristic Fano dip at $\lambda\approx900$ nm. Electric field distributions $\text{E}$ **d** and surface charge distributions $\rho$ **e** on the symmetry cutting plane at 890, 900, and 930 nm.

## 2.3 Field and Charge Distributions of IR806/AuNRs

The AuNRs (Fig. S3a and b) have a length of $L_{\mathrm{AuNR}}=\text{51 nm}$ and a radius of $R_{\mathrm{AuNR}}=\text{7.5}\text{ nm}$, capped with hemispherical ends. The AuNRs display a similar spectral response with AuNBPs (Fig. S3c). At 890 nm and 930 nm, the charges in the core and shell remain largely in phase, with the entire dipole moment flipping sign across the spectral maximum as in the AuNBPs. At 900 nm a partial phase delay appears between the two layers, yet the coupling region extends over a broader bandwidth (Fig. S3d and e).

**Fig. S3.** **Fano resonance of cylindrical AuNRs coated by 2-nm dye layer.** Geometries **a** and size parameters **b** of the AuNRs. **c** Extinction cross section of the hybrid Au@dye nanorod. Electric field distributions $\text{E}$ **d** and surface charge distributions $\rho$ **e** on the symmetry cutting plane at 890, 900, and 930 nm.

## 2.4 Comparison of Field and Charge Distributions

A side‐by‐side inspection highlights the geometric origin of the contrasting behaviors. The sharp tips of the bipyramid tightly confine the dipolar mode, causing an abrupt core–shell phase slippage that produces a distinct three‐stage sequence: in‐phase (890 nm) $\to$ phase‐mixed (900 nm) $\to$ inverted in‐phase (930 nm). In contrast, the curvature of the nanorod spreads the plasmon current over a larger volume. When the same phase inversion occurs, it proceeds more gradually and spans a wider spectral window, thereby eliminating the far‐field interference signature.

**2.5 Plasmon–Molecule Coupling at the Tip Hot Spots**

The molecular layers within the plasmonic hot spots serve as the physical origin of mode splitting, with their local intensity directly governing the magnitude of the splitting. To confirm the origin of mode splitting, the molecular layer with a height of 20 nm is selectively removed from the tip regions of AuNBPs ($\lambda_{\mathrm{LSPR}}=\text{90}\text{4}\text{ nm}$). The calculated extinction spectrum shows negligible splitting (Fig. S4a), and Fig. S4b and c confirm electric field and charge confinement, respectively. These results demonstrate that plasmon–molecule coupling at the tip hot spots is responsible for mode splitting; in the absence of molecules at the tips, such coupling is suppressed.

**Fig. S4. Effect of molecular location on optical responses.** **a** Extinction spectrum with no mode splitting after removing the molecules from the tips of AuNBPs (a height of 20 nm). **b, c** Corresponding electric field and charge distribution. These results confirm that the coupling is primarily localized at the tips, and the absence of molecules at the tips weakens the coupling, preventing mode splitting.

# Supplementary Note 3. Spectral Characterization and Resonance Hybridization

## 3.1 Configurations of Assembled IR806 Molecules

The characteristic extinction spectra of IR806 aqueous solutions at different concentrations are shown in Fig. S5a. These spectra exhibit two distinct peaks at 730 nm and 800 nm. The intensity ratio of these two absorption peaks essentially remains unchanged as the molecular concentration increases. The absorption peak at 800 nm corresponds to the monomer exciton resonance, whereas the absorption peak at 730 nm is caused by molecular vibrations. With the assistance of PAH, the assembled IR806 molecules exhibit a new absorption peak at 860 nm, as shown in Fig. S5b. The 860 nm absorption peak is redshifted relative to that of the monomer. This redshift suggests that this peak corresponds to J-aggregated dimers. The intensity ratio of the two absorption peaks at 730 nm and 800 nm gradually changes with increasing molecular concentration. The 730 nm absorption peak gradually increases in intensity, and its wavelength is blueshifted relative to that of the monomer, indicating that this peak corresponds to H-aggregated dimers. In addition, H-aggregates appear at approximately 700 nm, whereas J-aggregates arise at approximately 910 nm. H-aggregates are caused by tight molecular packing. J-aggregates are formed when the molecules align in a head-to-tail configuration.

**Fig. S5.** **a** Extinction spectra of IR806 aqueous solutions with different concentrations. **b** Extinction spectra of IR806 with different concentrations dissolved in the 10 mg·mL^-1^ PAH solution.

These five excitonic states have different configurations (the schematic at the top of Fig. S5b). The monomers adopt parallel or perpendicular orientations with respect to the substrate surface. Dimers, composed of two molecules oriented perpendicular to the surface, are categorized into H-aggregate dimers and J-aggregate dimers. Aggregates composed of multiple molecules aligned parallel to the surface are categorized into H-aggregates and J-aggregates depending on their stacking geometry. When the stacking direction of the IR806 molecules is perpendicular to their transition dipole moments, H-aggregates form; when it is parallel, J-aggregates form.

## 3.2 Concentration-Dependent Hybridized Resonances of IR806/AuNBP Hybrids with Different Values of $\boldsymbol{\lambda}_{\mathbf{LSPR}}$

The concentration-dependent extinction spectra of IR806/AuNBPs with different values of $\lambda_{\mathrm{LSPR}}$ are shown in Fig. S6. When $\lambda_{\mathrm{LSPR}}=\text{712 nm}$ and $\lambda_{\mathrm{LSPR}}=\text{778 }\text{nm}$, the intensity of the hybridized peaks near $\lambda_{\mathrm{LSPR}}$ decreases with increasing IR806 concentration. In contrast, the hybridized peak at approximately 910 nm becomes stronger as the IR806 concentration increases. Notably, no distinct Fano dip is observed in the extinction spectra under these conditions. However, when $\lambda_{\mathrm{LSPR}}=\text{870 nm}$, $\text{888 nm}$, $\text{904}\text{ nm}$, and $\text{942}\text{ nm}$, a clear Fano dip consistently appears near 910 nm in the extinction spectra. The reduction in extinction intensity at the Fano dip of the IR806/AuNBPs originates from coherent destructive interference between the J-aggregate excitons (formed by self-assembled IR806 molecules) and the plasmons, which implies a plasmon resonance energy transfer (PRET) process within the hybrids.

**Fig. S6.** Extinction spectra of IR806/AuNBPs as a function of the IR806 concentration at $\lambda_{\mathrm{LSPR}}=\text{712 nm}$ **a**, $\lambda_{\mathrm{LSPR}}=\text{778 nm}$ **b**, $\lambda_{\mathrm{LSPR}}=\text{8}\text{7}\text{0}\text{ nm}$ **c**, $\lambda_{\mathrm{LSPR}}=\text{8}\text{88}\text{ nm}$ **d**, $\lambda_{\mathrm{LSPR}}=\text{904}\text{ nm}$ **e**, and $\lambda_{\mathrm{LSPR}}=\text{9}\text{42}\text{ nm}$ **f**.

Figure S7 exhibits the concentration-dependent extinction spectra of IR806/AuNRs with different $\lambda_{\mathrm{LSPR}}$. As the concentration of IR806 molecules gradually increases, the extinction spectra of IR806/AuNRs undergoes significantly changes. For the IR806/AuNRs with $\lambda_{\mathrm{LSPR}}$ of 736 nm, 782 nm, and 810 nm, four hybridized absorption peaks and three distinct Fano dips are clearly observed, located near 772 nm, 820 nm, and 902 nm respectively. The Fano dip at 772 nm mainly arises from Fano interference superposition of plasmons coupling with D_h_ excitons. The Fano dip at 820 nm is due to the Fano interference superposition of plasmons coupling with D_j_ excitons. The Fano dip at 902 nm is mainly the result of Fano interference between J-aggregate excitons and plasmons. When the LSPR wavelength is 872 nm, a Fano dip appears at 772 nm, while a broader and more obvious Fano dip emerges near 822 nm. This broad Fano dip results from the combined plasmon coupling with multiple excitons, including J-aggregate excitons.

**Fig. S7.** Extinction spectra of the IR806/AuNRs as a function of the IR806 concentration at $\lambda_{\mathrm{LSPR}}=\text{7}\text{36}\text{ nm}$ **a**, $\lambda_{\mathrm{LSPR}}=\text{7}\text{82}\text{ nm}$ **b**, $\lambda_{\mathrm{LSPR}}=\text{8}\text{10}\text{ nm}$ **c**, and $\lambda_{\mathrm{LSPR}}=\text{87}\text{2}\text{ nm}$ **d**.

## 3.3 Peak Fitting of the Extinction Spectra of IR806/AuNBPs

In our study, peak fitting is performed on the extinction spectra of IR806/AuNBPs at a dye concentration of 2 $\mu mol\cdot L^{-1}$ to resolve overlapping spectral features. The spectra are fitted using five Lorentzian functions (denoted as Peaks 2–6) together with a Fano lineshape (denoted as Peak 1), as shown in Fig. S8. Obvious changes are observed in the multiple hybridized peaks as the plasmon resonance wavelength of the AuNBPs increases.

**Fig. S8.** Peak fittings of extinction spectra for the IR806/AuNBPs with $\lambda_{\mathrm{LSPR}}$ at 736 nm **a**, 844 nm **b**, 870 nm **c**, and 904 nm **d**. The main contribution to the hybrid spectrum intensity comes from Peak 5 for $\lambda_{\mathrm{LSPR}}=\text{736 nm}$ in **a**, Peak 3 for $\lambda_{\mathrm{LSPR}}=\text{844}\text{ nm}$ in **b**, Peak 2 for $\lambda_{\mathrm{LSPR}}=\text{870 n}m$ in **c**, and Peak 1 for $\lambda_{\mathrm{LSPR}}=\text{904 nm}$ in **d**.

## 3.4 Peak Fitting of the Extinction Spectra of IR806/AuNRs

Similarly, the extinction spectra of IR806/AuNRs at a dye concentration of 2 $\mu mol\cdot L^{-1}$ are fitted using a combination of Lorentzian and Fano lineshapes to resolve overlapping spectral features (Fig. S9). Obvious changes are also observed in the multiple hybridized peaks as the plasmon resonance wavelength of AuNRs increases.

**Fig. S9.** Four representative peak fittings of extinction spectra for the IR806/AuNRs with $\text{λ}_{\text{LSPR}}$ at 736 nm **a**, 794 nm **b**, 854 nm **c**, and 888 nm **d**. The main contribution to the hybrid spectrum intensity comes from Peak 5 for $\text{λ}_{\text{LSPR}}=736 nm$in **a**, Peak 5 for $\text{λ}_{\text{LSPR}}=794 nm$ in **b**, Peak 2 for $\text{λ}_{\text{LSPR}}=854 nm$ in **c**, and Peak 2 for $\text{λ}_{\text{LSPR}}=888 nm$ in **d**.
**Supplementary Note 4. Tuning Multiple Fano Interferences via Molecular Configuration and Orientation Control**

## 4.1 Coupled Harmonic Oscillator Model of IR806/AuNBPs and IR806/AuNRs

The plasmon–exciton coupling can be described by a classical coupled harmonic oscillator model with $2\times2$ Hamiltonian

$$\begin{aligned} H=\left( \begin{matrix} E_{p}-\frac{{i\gamma}_{p}}{2} & \Delta\\ \Delta& E_{c}-\frac{{i\gamma}_{c}}{2} \end{matrix} \right)\#\text{(S4)} \end{aligned}$$

where $E_{p}$ ($\gamma_{p}$) and $E_{c}$ ($\gamma_{c}$) represent the energies (decay rates) of plasmon and exciton, respectively. The two hybridized modes can be expressed as

$$\begin{aligned} E_{\pm}=\frac{1}{2}\left( E_{p}+E_{c} \right)-\frac{i}{4}\left( \gamma_{p}+\gamma_{c} \right)\pm\frac{1}{2}\sqrt{\left( 2\Delta\right)^{2}+\left[ \left( E_{p}-E_{c} \right)+\frac{i}{2}\left( \gamma_{p}-\gamma_{c} \right) \right]^{2}}\#(S5) \end{aligned}$$

where $2\Delta=2g$ is the Rabi splitting constant, and $g$ is coupling strength^4–6^. There are two criterions that are commonly used to discuss the strong coupling of hybrids with Rabi splitting:

i) $2\Delta=2g>\frac{1}{2}\left( \gamma_{p}-\gamma_{c} \right)$, it guarantees two real solutions for $E_{\pm}$ in the coupled-oscillator model when the detuning is zero ($E_{p}=E_{c}$). It does not ensure the observation of Rabi splitting, as the two hybridized states at zero detuning exhibit a linewidth characterized by $\frac{1}{2}\left( \gamma_{p}+\gamma_{c} \right)$.

ii) $2\Delta=2g>\frac{1}{2}\left( \gamma_{p}+\gamma_{c} \right)$, it guarantees that at least one complete Rabi oscillation occurs, rather than the occurrence of Fano interference with energy transfer at Fano dip in the intermediate coupling regime. The Rabi splitting will be experimentally visible when the difference of two hybridized states is bigger than the linewidths of the new modes. This criterion is sometimes given also as $\left( 2g \right)^{2}>\frac{1}{2}\left( \gamma_{p}^{2}+\gamma_{c}^{2} \right)$, and it is obviously the same as $2g>\frac{1}{2}\left( \gamma_{p}+\gamma_{c} \right)$ if $\gamma_{p}\sim\gamma_{c}$^4–5^.

When the decay rates of plasmon ($\gamma_{p}$) and exciton ($\gamma_{c}$) are much smaller than their energies, the two hybridized modes can be expressed as^4^

$$\begin{aligned} E_{\pm}=\frac{1}{2}\left( E_{p}+E_{c} \right)\pm\frac{1}{2}\sqrt{\left( 2\Delta\right)^{2}+\left( E_{p}-E_{c} \right)^{2}}\#\text{(S6)} \end{aligned}$$

The coupled-oscillator Hamiltonian for the calculation of energy splitting of hybridized states can be used to describe both Rabi splitting and Fano interference when $2\Delta=2g>\frac{1}{2}\left( \gamma_{p}-\gamma_{c} \right)$, and the transition from Rabi splitting to Fano interference can be achieved by tuning the coupling strength or the decay rate of plasmon/exciton in the coupling system^7,8^. We use this coupled-oscillator model to analyze the hybridization of one plasmon resonance and five exciton resonances in the Fano regime.

## 4.2 IR806 Molecular Orientation on AuNBPs and AuNRs

Our calculations demonstrate two distinct behaviors of exciton–plasmon coupling within the IR806/AuNR and IR806/AuNBP hybrids. The IR806/AuNRs exhibit considerably stronger coupling with D_h_ and D_j_ excitons, with coupling constants that are 23% and 22% greater, respectively, than those of the IR806/AuNBPs (Fig. S10). This pronounced enhancement indicates that plasmon interactions with D_h_ and D_j_ excitons dominate for the IR806/AuNRs. Conversely, compared with the IR806/AuNRs, the IR806/AuNBPs displays a 10% greater coupling constant for J-aggregate excitons, revealing that J-aggregate exciton‒plasmon coupling is the predominant interaction for the IR806/AuNBP hybrids.

**Fig. S10.** **a** Coupling constants of plasmon with five excitons (H, D_h_, M, D_j_, and J) of IR806/AuNRs and IR806/AuNBPs. **b** Configuration and orientation of IR806 molecules attached to the surfaces of AuNRs and AuNBPs.

The coupling constant between plasmons and molecules can be expressed as

$$\begin{aligned} g_{\mathrm{pc}}=\frac{\hbar\omega}{\sqrt{2\hbar\varepsilon_{0}\omega_{d}V_{eff}}}\boldsymbol{\mu}_{c}\cdot{\hat{\boldsymbol{f}}}_{p}\left( \boldsymbol{r}_{c} \right)\#\text{(S7)} \end{aligned}$$

Where $V_{\mathrm{eff}}=V_{m}\times n^{2}\left( \boldsymbol{r} \right)$ is the effective mode volume of the plasmon mode; $V_{m}$ is the actual mode volume; $n\left( \boldsymbol{r} \right)$ is the refractive index at $\boldsymbol{r}$; $\boldsymbol{\mu}_{c}$ and $\boldsymbol{r}_{c}$ are the dipole moment and location of the exciton, respectively; ${\hat{\boldsymbol{f}}}_{p}\left( \boldsymbol{r}_{c} \right)=\boldsymbol{E}_{p}\left( \boldsymbol{r} \right)/\left| \boldsymbol{E}_{p}\left( \boldsymbol{r} \right) \right|_{\max}$ is the normalized electric field (EF) of the plasmon mode^5,9^.

## Briefly, the coupling constant is positively correlated with $\boldsymbol{\mu}_{\mathbf{c}}\boldsymbol{\cdot}{\hat{\boldsymbol{f}}}_{\mathbf{p}}\left( \boldsymbol{r}_{\mathbf{c}} \right)\boldsymbol{=}\boldsymbol{\mu}_{\mathbf{c}}\cos\boldsymbol{\theta}$, where $\boldsymbol{\theta}$ denotes the angle between the molecular dipole moment and the plasmonic electric field. In the IR806/AuNBP (or IR806/AuNR) hybrids, the coupling constant is related to the dot product of the molecular dipole moment and the localized electric field, or equivalently, the cosine of the angle between them.

## 4.3 Aggregation Forms of Assembled IR806 Molecules Dependent on the LSPR Wavelength

Unlike the aggregation form of assembled IR806 molecules under the influence of PAH, the aggregation forms of assembled IR806 molecules are also affected by the plasmon resonance wavelength. In Fig. S11, the extinction spectra of assembled IR806 molecules on nanomaterials is obtained by subtracting the extinction spectra of two sets of high-concentration IR806/AuNBPs that are already in a saturated state. As $\lambda_{\mathrm{LSPR}}$ increases, the intensity of the J-aggregate and H-aggregate resonance peaks relative to the monomer resonance peak increases, indicating that more J-aggregate and H-aggregate excitons are undergoing Fano interference with the plasmons in the IR806/AuNBP hybrids. Particularly, when $\lambda_{\mathrm{LSPR}}=\text{904}\text{ nm}$, the intensity ratio of J-aggregates to monomers reaches its maximum, which also explains why the resonance energy transfer between IR806 excitons and plasmons is strongest under these conditions.

**Fig. S11.** Extinction spectra of assembled IR806 molecules on AuNBPs with different values of $\lambda_{\mathrm{LSPR}}$.

# Supplementary Note 5. Ultrafast Transient Absorption and Z-Scan Nonlinear Transmittance Measurements

## 5.1 Ultrafast Pump-Probe Measurements

According to the absorption law, the absorbance $A=-\lg T$, where *T* is the transmittance. In pump-probe measurements, the transient differential absorbance is obtained by comparing the probe transmission with and without pump pulse excitation^10^:

$$\begin{aligned} \Delta A=A_{pump on}-A_{pump off}\#\text{(S}\text{8}\text{)} \end{aligned}$$

$$\begin{aligned} \Delta A=-lg\frac{I_{\text{pump on}}}{I}-\left( -lg\frac{I_{\text{pump off}}}{I} \right) \\ =-\lg\frac{I_{\text{pump on}}}{I_{pump off}}=-lg\frac{\Delta I+I_{pump off}}{I_{pump off}}=-lg(\frac{\Delta I}{I_{0}}+1)\#\text{(S}\text{9}\text{)} \end{aligned}$$

where $I_{pump off}$ denotes the probe-transmitted light intensity of the nonexcited sample (defined as $I_{0}$); $I_{pump on}$ represents the probe-transmitted light intensity of the excited sample, and $\Delta I$ is the difference between $I_{pump on}$ and $I_{pump off}$. The measured transient differential absorbance $\Delta A\left( t,\lambda\right)$ is a function of delay time (the interval between the pump and the probe pulses) and probe wavelength.

## 5.2 Z-Scan Measurements

The nonlinear absorptions (NLA) of the bare AuNBPs, bare IR806 (PAH) molecules and IR806/AuNBP hybrids have been investigated via the open-aperture (OP) Z-scan technique using a femtosecond-pulsed Ti:sapphire lasers (150 fs, 76 MHz, Mira 900 Coherent). The solution sample into a quartz cuvette with a thickness of $L=1 \mathrm{mm}$. The power-dependent absorption coefficient can be written as

$$\begin{aligned} \alpha\left( I \right)=\alpha_{0}+\beta_{\mathrm{eff}}I\#\text{(S}\text{10}\text{)} \end{aligned}$$

with the linear coefficient $\alpha_{0}$ and the nonlinear part absorbed in the function $\beta_{\mathrm{eff}}I$. The intensity-dependent absorption of the saturated effect and its first-order Taylor expansion is

$$\begin{aligned} \alpha\left( I \right)=\frac{\alpha_{0}}{1+\frac{I}{I_{S}}}\approx\alpha_{0}\left( 1-\frac{I}{I_{S}} \right)\#\text{(S}\text{11}\text{)} \end{aligned}$$

where $I_{S}$ is the saturated intensity ($I_{S,p}$ or $I_{S,i}$). Combining Equations S10 and S11, the formula for the saturation intensity is obtained as

$$\begin{aligned} I_{S}\approx\frac{{-\alpha}_{0}}{\beta_{\mathrm{eff}}}\#\text{(S}\text{12}\text{)} \end{aligned}$$

In the Z-scan theory, the effective NLA coefficient $\beta_{\mathrm{eff}}$ can be calculated by open-aperture transmittance $T_{\mathrm{OP}}$ as following relationships^11^:

$$\begin{aligned} T_{\mathrm{OP}}=\sum_{m=0}^{\infty} \left( \frac{\left( -q_{0} \right)^{m}}{\left( 1+{z^{2}}/{z_{0}^{2}} \right)^{m}\left( 1+m \right)^{3/2}} \right)\#\text{(S13)} \end{aligned}$$

with $q_{0}=\beta_{\mathrm{eff}}I_{0}L_{\mathrm{eff}}$, where $I_{0}$ is the peak irradiance at the focus ($z=0$), and $L_{\mathrm{eff}}=[1-\exp(-\alpha_{0}L)]/\alpha_{0}$ is the effective thickness of the sample. Expand the formula and take the first approximation. The normalized $T_{\mathrm{OP}}$ follows the relation$T_{\mathrm{OP}}\left( z \right)=1-I\left( z \right)\beta_{\mathrm{eff}}L_{\mathrm{eff}}$, where $I(z)=I_{0}/[1+{(z/z_{0})}^{2}]$ is the light intensity of a Gaussian laser beam at the position $z$, and $z_{0}$ is effective Rayleigh length at the focus ($z=0$) of the beam. When we consider only the second order nonlinear process with a constant $\beta_{\mathrm{eff}}$, the measured $T_{\mathrm{OP}}$ can be fitted with a Lorentz function of $z$. We selected a laser wavelength of 904 nm, the resonance wavelength of bare AuNBPs, with a power of 15 mW ($I_{0}=0.469\mathrm{GW}\cdot\mathrm{cm}^{-2}$).

## 5.3 Transient Differential Absorption Spectra of Bare AuNRs and AuNBPs with Different Values of $\boldsymbol{\lambda}_{\mathbf{LSPR}}$

In transient absorption measurements, the saturable absorption (SA) of bare AuNRs decreases with increasing $\lambda_{\mathrm{LSPR}}$, whereas longer nanorods exhibit an extended plasmon lifetime and a slower hot electron relaxation dynamics^12^. As shown in Fig. S12a, the bare AuNBPs with different $\lambda_{\mathrm{LSPR}}$ display SA when excited at their respective plasmon resonance wavelengths and therefore do not contribute to the reverse saturable absorption (RSA) observed in the IR806/AuNBPs. The intensity of $\Delta A_{m}$ tends to decrease as $\lambda_{\mathrm{LSPR}}$ of bare AuNBPs increases, although the relationship is not strictly linear, as illustrated in Fig. S12b.

**Fig. S12.** **Measured differential absorption magnitude** $\boldsymbol{\Delta}\boldsymbol{A}\left( \boldsymbol{t} \right)$ **of bare AuNBPs with different values of** $\boldsymbol{\lambda}_{\mathbf{LSPR}}$ **at various excitation wavelengths.** **a** Transient decay spectra of AuNBPs with different values of $\lambda_{\mathrm{LSPR}}$ measured under excitation at their respective plasmon resonance wavelengths. **b** Variation in the peak intensity of nonlinear absorption $\Delta A_{m}$ with respect to $\lambda_{\mathrm{LSPR}}$.

## 5.4 Transient Differential Absorption Spectra of Bare IR806 Molecules

We measured the transient differential absorption of bare IR806 molecules (PAH) with a concentration of 2 $\mu mol\cdot L^{-1}$, which is the maximal molecular concentration in the plasmon–exciton hybrids. At an excitation fluence of $I_{\mathrm{pump}}=9.3 \mu J\cdot\mathrm{cm}^{-2}$, the signal of SA is very weak (comparable with noise) across all five exciton resonance wavelengths (Fig. S13). This indicates that the NLA effect, such as excited-state absorption (ESA), is not observable in the IR806 molecules.

**Fig. S13. Transient Differential Absorption of IR806 molecules measured at various excitation wavelengths.** The spectra are vertically shifted for clarity.

## 5.5 Power-Dependent Transient Differential Absorption Spectra of Bare IR806 Molecules, Bare AuNBPs, and IR806/AuNBPs

The IR806/AuNBP hybrids shown in Fig. S14a are prepared by assembling 904-nm AuNBPs with 2 $\mu mol\cdot L^{-1}$ IR806 molecules. The bare IR806 molecules with PAH, AuNBPs, and IR806/AuNBP hybrids were subjected to power-dependent measurements with a 904-nm excitation under identical experimental conditions. For the bare 2 $\mu mol\cdot L^{-1}$ IR806 molecules with PAH, no transient signal is observed in the power-dependent measurements (Fig. S14b). For the bare AuNBPs, SA is observed when the pump fluence increases to 3.10 $\mu J\cdot\mathrm{cm}^{-2}$, which originates from the ground state bleaching (GSB) of AuNBPs under resonant excitation. The SA signal increases linearly with pump fluence increasing, and no saturation effect of SA is observed. The maximum of SA is still not reached even at a pump fluence of $55.8 \mu J\cdot\mathrm{cm}^{-2}$ (Fig. S14c and d).

For the IR806/AuNBP hybrids, RSA is still present at low pump fluence. The RSA signal increases rapidly with pump fluence; however, this growth slows down significantly when the pump fluence exceeds $9.3 \mu J\cdot\mathrm{cm}^{-2}$ (Fig. S14e and f). This behavior may arise from the saturation of the unidirectional energy transfer between AuNBPs and IR806 molecules.

**Fig. S14. Power-dependent transient differential absorption spectra of bare IR806 molecules, bare AuNBPs, and IR806/AuNBPs.** **a** Extinction spectra of IR806 molecules with PAH, AuNBPs, and the IR806/AuNBP hybrids ($\lambda_{\mathrm{LSPR}}=904 \mathrm{nm}$). **b, d, f** Power-dependent transient decay spectra of molecules with PAH, AuNBPs, and IR806/AuNBPs, respectively (spectra vertically shifted for clarity). **c, e** $\Delta A$ as a function of pump fluence for AuNBPs (**c**) and IR806/AuNBPs (**e**).

## 5.6 Transient Differential Absorption Spectra of IR806/AuNBPs and Bare AuNBPs with $\boldsymbol{\lambda}_{\mathbf{LSPR}}\mathbf{=820 and 904 nm}$

For the IR806/AuNBPs with $\lambda_{\mathrm{LSPR}}=\text{820 }\text{nm}$, as the excitation wavelength increases, SA is first observed, followed by reverse RSA. The differential absorbance magnitude $\Delta A$ of RSA reaches its maximum at $\lambda_{\mathrm{exc}}=\text{82}\text{0 nm}$, and a second RSA peak is detected at $\lambda_{\mathrm{exc}}=\text{89}\text{5}\text{ nm}$. Beyond this point, a transition from RSA back to SA is observed (Fig. S15a). In contrast, the bare AuNBPs primarily exhibit SA under different excitation wavelengths, and $\Delta A$ of SA reaches its maximum at $\lambda_{\mathrm{exc}}=\text{820 nm}$ (Fig. S15b). When the $\lambda_{e\mathrm{xc}}$ exceeds the $\lambda_{\mathrm{LSPR}}$, weak RSA occurs, likely due to the structural characteristics of AuNBPs and surface plasmon potential. For the IR806/AuNBPs hybrids with $\lambda_{\mathrm{LSPR}}=\text{904}$ nm, we observe remarkably strong RSA under different $\lambda_{e\mathrm{xc}}$, reaching a maximum $\Delta A$ of 2.38 mOD at the $\lambda_{e\mathrm{xc}}=910 nm$ (Fig. S15c). In Fig. S15d, the bare AuNBPs consistently exhibit SA across all excitation wavelengths. The maximum of SA of bare AuNBPs is observed at $\lambda_{\mathrm{exc}}=\text{900 nm}$, with $\Delta A$ of $0.16 mOD$.

**Fig. S15.** **Differential absorption magnitude** $\boldsymbol{\Delta}\boldsymbol{A}\left( \boldsymbol{t} \right)$ **of IR806/AuNBPs and bare AuNBPs with** $\boldsymbol{\lambda}_{\mathbf{LSPR}}\boldsymbol{=}\text{820}$ **nm and** $\boldsymbol{\lambda}_{\mathbf{LSPR}}\boldsymbol{=}\text{904}$ **nm.** Transient decay spectra of IR806/AuNBPs ($\lambda_{\mathrm{LSPR}}=\text{820}$ nm) recorded with excitation wavelength across 700–950 nm **a**, bare AuNBPs ($\lambda_{\mathrm{LSPR}}=820$ nm) across 720–890 nm **b**, IR806/AuNBPs ($\lambda_{\mathrm{LSPR}}=\text{904}$ nm) across 700–990 nm **c**, and bare AuNBPs with $\lambda_{\mathrm{LSPR}}=\text{904 nm}$ across 750–940 nm **d**.

## 5.7 Open-Aperture Z-Scan Nonlinear Transmittance of Bare IR806 Molecules, Bare AuNBPs, and IR806/AuNBP Hybrids

In the Z-scan measurements, 2 $\mu mol\cdot L^{-1}$ IR806 molecules with PAH exhibit SA under an excitation at J-exciton resonance wavelength of 904 nm (Fig. S16). The nonlinear coefficient is calculated as $\beta_{eff,i=J}=-0.727 \mathrm{cm}\cdot\mathrm{GW}^{-1}$, and the corresponding saturated intensity is $I_{S,i=J}\approx-\frac{\alpha_{0,i=J}}{\beta_{eff,i=J}}=0.058 \mathrm{GW}\cdot\mathrm{cm}^{-2}$.

The bare AuNBPs ($\lambda_{\mathrm{LSPR}}=\text{904}$ nm) also exhibit SA signal due to the bleaching of the electronic ground state, which is more prominent at the plasmon resonance wavelength owing to the stronger local field enhancement^13^. Calculations from the Z-scan experimental data obtain $\beta_{eff,p}=-0.95 cm\cdot\mathrm{GW}^{-1}$ and $I_{S,p}\approx-\frac{\alpha_{0,p}}{\beta_{eff,p}}=2.43 GW\cdot\mathrm{cm}^{-2}$ ($I_{S,p}=5.18I_{0}$). The saturated intensity of bare AuNBPs is approximately two-order of magnitude larger than that of the bare IR806 molecules. These results are consistent with the saturated intensity measured in similar Au nanostructures^14^.

The IR806/AuNBP hybrids show a reduced transmittance at the focus ($z=0$), corresponding to RSA, with $\beta_{\mathrm{eff}}=+2.55 cm\cdot\mathrm{GW}^{-1}$. This behavior is independent of the individual components and instead arises from the PRET induced by their coupling.

**Fig. S16. Nonlinear transmittance of bare IR806 molecules with PAH, bare AuNBPs, and IR806/AuNBP hybrids.** The laser power for the three samples is fixed at $P_{\mathrm{laser}}=15 \mathrm{mW}$ ($I_{0}=0.469\mathrm{GW}\cdot\mathrm{cm}^{-2}$). The normalized Z-scan transmittance curves are vertically shifted for clarity. The reduced transmittance observed in the IR806/AuNBPs hybrid is distinct from the responses of both the bare AuNBPs and the IR806 molecules with PAH.

## 5.8 Fano Lineshape Fitting of Nonlinear Absorption Spectra

The linear absorption spectrum shows a characteristic Fano profile induced by Fano interference, whereas the nonlinear absorption spectrum presents an inverse profile. Alternatively, both spectra can be fitted simultaneously using two Fano lineshapes. By defining a reduced absorption by $\epsilon=\frac{E-E_{0}}{\Gamma_{E}/2}\approx\frac{\lambda-\lambda_{0}}{\Gamma_{\lambda}/2}$, the Fano profile^15^ can be expressed as follows:

$$\begin{aligned} \sigma\left( \lambda\right)=a\frac{\left( q+\epsilon\right)^{2}}{1+\epsilon^{2}}+b=a\frac{{[q+2(\lambda-\lambda_{0})/\Gamma_{\lambda}]}^{2}}{1+{[2(\lambda-\lambda_{0})/\Gamma_{\lambda}]}^{2}}+b\#\text{(S14)} \end{aligned}$$

where $q$ is the Fano parameter, which describes the degree of asymmetry in the Fano lineshape; $q=\cot\delta$; $\delta$ represents the phase shift between the plasmon and exciton; $\lambda_{0}$ is the resonance peak wavelength; $\Gamma_{\lambda}$ denotes the full width at half maximum (FWHM) of the resonance peak; $a$ is the amplitude factor; and $b$ is the baseline offset. The Fano lineshape profiles are shown in Fig. S17. The Fano parameter $q$ of Fano fitting $I$ is consistently negative, whereas that of Fano fitting $\text{Ⅱ}$ is always positive. With increasing $\lambda_{\mathrm{LSPR}}$, the phase shift between the plasmons and excitons gradually decreases. When the two peaks of the Fano profile coincide at $\lambda_{\mathrm{LSPR}}=\text{904}$ nm, the nonlinear absorption reaches its maximum value.

**Fig. S17.** **Fano lineshape fitting for the nonlinear absorption of IR806/AuNBPs with** $\boldsymbol{\lambda}_{\mathbf{LSPR}}\mathbf{=}$**778, 844, and 912 nm.** **a** For the hybrids with $\lambda_{\mathrm{LSPR}}=778$ nm, $q_{\text{Ⅰ}}=-0.71$ and $q_{\text{Ⅱ}}=1.91$. **b** For the hybrids with $\lambda_{\mathrm{LSPR}}=844$ nm, $q_{\text{Ⅰ}}=-2.2$ and $q_{\text{Ⅱ}}=2.4$. **c** For the hybrids with $\lambda_{\mathrm{LSPR}}=912$ nm, $q_{\text{Ⅰ}}=-1.8$ and $q_{\text{Ⅱ}}=0.9$.

## 5.9 Transient Differential Absorption Spectra of IR806/AuNBPs with Different Values of $\boldsymbol{\lambda}_{\mathbf{LSPR}}$

The IR806/AuNBPs with different plasmon resonances ($\lambda_{\mathrm{LSPR}}=\text{696–942}$ nm) exhibit their maximum RSA when the $\lambda_{\mathrm{exc}}$ is close to $\lambda_{\mathrm{LSPR}}$. Notably, for the IR806/AuNBPs with $\lambda_{\mathrm{LSPR}}=\text{928}$ nm and $\lambda_{\mathrm{LSPR}}=\text{942}$ nm, the optimal RSA excitation wavelength remains fixed at 920 nm, demonstrating a decoupling from further LSPR redshift (Fig. S18).

**Fig. S18.** **Differential absorption magnitude** $\boldsymbol{\Delta}\boldsymbol{A}\left( \boldsymbol{t} \right)$ **of IR806/AuNBPs with different values of** $\boldsymbol{\lambda}_{\mathbf{LSPR}}$**.** Maximum magnitude $\Delta A_{m}$ of transient absorption of IR806/AuNBPs with $\lambda_{\mathrm{LSPR}}=736\mathrm{nm}$ **a**, 778 nm **b**, and 844 nm **c** at different $\lambda_{\mathrm{exc}}$. **d** Transient decay spectra of IR806/AuNBPs with different values of $\lambda_{\mathrm{LSPR}}$ at the maximum RSA effect.

## 5.10 Transient Differential Absorption Spectra of IR806/AuNRs with $\boldsymbol{\lambda}_{\mathbf{LSPR}}\mathbf{=782 nm, 820 nm, and 872 nm}$.

The nonlinear absorption spectrum of IR806/AuNRs ($\lambda_{\mathrm{LSPR}}=\text{782}$ nm) exhibits three RSA peaks, which is different from the IR806/AuNBPs. The three RSA peaks (PRET peak *A*, *B*, and *C*) correspond directly to the three Fano dips observed in the linear absorption spectra (Fig. S19a and b). For the IR806/AuNRs with $\lambda_{\mathrm{LSPR}}=\text{820 nm}$, the nonlinear response evolves as the excitation wavelength increases: SA occurs first, followed by RSA with three peaks, and finally transition back to SA, resulting in a characteristic W-shaped spectrum. The differential absorption magnitude $\Delta A$ of RSA reaches its maximum at $\lambda_{\mathrm{exc}}=\text{840}\text{ nm}$. Specifically, the three RSA peak can be assigned to specific exciton–plasmon couplings: the peak at $\lambda_{\mathrm{exc}}=\text{900}$nm (labelled as $\lambda_{PRET,A}$) arises mainly from the coupling between plasmons and J excitons; the peak at $\lambda_{\mathrm{exc}}=\text{840}$nm (labelled as $\lambda_{PRET,\text{B}}$) is primarily induced by the coupling between plasmons and D_j_ excitons; and the peak at $\lambda_{\mathrm{exc}}=\text{790}$nm (labelled as $\lambda_{PRET,\text{C}}$) is due to the coupling between plasmons and D_h_ excitons(Fig. S19c and d). For the IR806/AuNRs with $\lambda_{\mathrm{LSPR}}=\text{872}$ nm, the RSA peaks are overlapped, and the original W-lineshape disappears. The PRET efficiency ($\lambda_{\mathrm{LSPR}}=872$ nm, $\Delta A_{m}=$1.12 mOD) remains lower than that of the IR806/AuNBPs ($\lambda_{\mathrm{LSPR}}=\text{870}$ nm, $\Delta A_{m}=$1.87 mOD) (Fig. S19e and f).

**Fig. S19.** **Differential absorption magnitude of IR806/AuNRs.** Transient decay spectra of IR806/AuNRs with $\lambda_{\mathrm{LSPR}}=$782 nm **a**, 820 nm **b**, and 872 nm **c**. Maximum magnitude $\Delta A_{m}$ of transient absorption of IR806/AuNRs with $\lambda_{\mathrm{LSPR}}=$782 nm **d**, 820 nm **e**, and 872 nm **f** at different excitation wavelengths.

# Supplementary Note 6. Theory and Calculation of Linear and Nonlinear Absorption Spectra

To gain a deeper understanding of the optical response of the plasmon–exciton hybrid system, we develop a theoretical framework that combines linear and nonlinear effects.

## 6.1 Linear Absorption

For multiple plasmonic or excitonic modes without mutual interactions, the total response function can be expressed as a superposition of independent contributions:

$$\begin{aligned} \boldsymbol{\chi}^{0}=diag\left( \chi_{p},\chi_{1},\chi_{2},\chi_{3},\chi_{4},\chi_{5} \right)\#\text{(S}\text{15}\text{)} \end{aligned}$$

where $\chi_{p}$ and $\chi_{i}$ denote the response functions of the plasmons and molecular excitons, respectively. The plasmonic response function is written as follows:

$$\begin{aligned} \chi_{p}=-\frac{n_{p}}{\omega-\omega_{p}+i\gamma_{p}}\#\text{(S}\text{16}\text{)} \end{aligned}$$

where $\omega_{p}$ is the plasmon resonance frequency, $\gamma_{p}$ is the decay rate, and $n_{p}$ is the concentration factor. Similarly, the excitonic response function for the *i*-th molecular resonance is expressed as

$$\begin{aligned} \chi_{i}=-\frac{n_{i}}{\omega-\omega_{i}+i\gamma_{i}}\#\text{(S}\text{17}\text{)} \end{aligned}$$

where $\omega_{i}$, $\gamma_{i}$, and $n_{i}$ denote the resonance frequency, decay rate, and concentration of the *i*-th molecular state, respectively. By considering the interaction between the external field and the plasmon $V_{p}$ or molecular states $V_{i}$, we can write as follows:

$$\begin{aligned} \boldsymbol{V}_{\mathrm{ext}}=\left( \begin{matrix} V_{p} & V_{1} & V_{2} & V_{3} & V_{4} & V_{5} \end{matrix} \right)^{T}\#\text{(S1}\text{8}\text{)} \end{aligned}$$

and the absorption of the noninteracting system is

$$\begin{aligned} \sigma_{0}=-Im\left( \boldsymbol{V}_{\mathrm{ext}}^{\dagger} \boldsymbol{\chi}^{0} \boldsymbol{V}_{\mathrm{ext}} \right)\#\text{(S1}\text{9}\text{)} \end{aligned}$$

To account for plasmon–exciton coupling, we introduce the interaction term:

$$\begin{aligned} \boldsymbol{K}=\left( \begin{matrix} 0 & \boldsymbol{M}^{T} \\ \boldsymbol{M} & 0_{5\times5} \end{matrix} \right)\#\text{(S}\text{20}\text{)} \end{aligned}$$

with

$$\begin{aligned} \boldsymbol{M}=\left( \begin{matrix} M_{1} & M_{2} & M_{3} & M_{4} & M_{5} \end{matrix} \right)^{T}\#\text{(S}\text{21}\text{)} \end{aligned}$$

which characterizes the coupling strength between the plasmon mode and the *i*-th excitonic mode. By applying the Dyson equation to the coupled system, the renormalized response function is obtained as follows:

$$\begin{aligned} \boldsymbol{\chi}_{h}=\left( \boldsymbol{I}_{6\times6}-\boldsymbol{\chi}^{0}\boldsymbol{K} \right)^{-1}\boldsymbol{\chi}^{0}\#\text{(S}\text{22}\text{)} \end{aligned}$$

Eventually, the absorption spectrum of the hybrid system is given by

$$\begin{aligned} \sigma_{h}=-Im\left( \boldsymbol{V}_{\mathrm{ext}}^{\dagger} \boldsymbol{\chi}_{h} \boldsymbol{V}_{\mathrm{ext}} \right)\#\text{(S23)} \end{aligned}$$

## 6.2 Saturable Nonlinear Absorption

For saturable absorption, we start from the saturable absorption intensity, which can be described as follows:

$$\begin{aligned} I_{S}=\frac{\hbar\omega}{\sigma\tau}=\hbar\omega\frac{\gamma}{\sigma}\#\text{(S}\text{24}\text{)} \end{aligned}$$

Physically, this relation reflects the balance between the absorption rate $\sigma$ and the relaxation time $\tau$ (or dissipation $\gamma$) of the system. Notably, saturable nonlinear absorption is fundamentally different from the coherent second-order ($\chi^{\left( 2 \right)}$) or third-order ($\chi^{\left( 3 \right)}$) nonlinear processes typically discussed in nonlinear optics. In the latter case, the nonlinear response arises from coherent polarization processes. In contrast, saturable absorption originates from noncoherent population dynamics, where the ground state population is depleted at high intensities, leading to reduced absorption. In our framework, this effect is explicitly introduced through the concentration $\bar{n}_{i}$ associated with each molecular state, which governs the intensity dependence of the absorption as follows:

$$\begin{aligned} \bar{n}_{i}=\frac{n_{i}}{1+I_{0}/I_{S,i}}\#\text{(S}\text{25}\text{)} \end{aligned}$$

where

$$\begin{aligned} I_{S,i}=\hbar\omega\frac{\bar{\gamma}_{i}}{\sigma_{i}}\#\text{(S}\text{26}\text{)} \end{aligned}$$

Here, $\sigma_{i}$ indicates the absorption strength of the *i*-th molecular state and $I_{S,i}$ corresponds to its effective saturation intensity. The dissipation rate $\bar{\gamma}_{i}$ of each molecular state plays a key role here. $\bar{\gamma}_{i}$ differs from the intrinsic decay rate of an isolated molecule ($\gamma_{i}$), as it incorporates environmental effects and plasmon–molecule interactions. In this study, $\bar{\gamma}_{i}$ is treated as a tunable parameter to account for coupling effects and inhomogeneous broadening.

To explicitly evaluate the nonlinear absorption spectrum, we replace the molecular concentration $n_{i}$ in the hybrid absorption formula with the effective population number $\bar{n}_{i}$, which reflects the saturation-induced depletion of molecular states. The nonlinear absorption spectrum is then obtained by calculating the difference between the absorption with $\bar{n}_{i}$ and that with the original $n_{i}$:

$$\begin{aligned} \beta\left( \omega\right)= \sigma_{h}\left( \omega;\bar{n}_{i} \right)-\sigma_{h}\left( \omega;n_{i} \right)\#\text{(S}\text{2}\text{7}\text{)} \end{aligned}$$

It is important to note that the nonlinear absorption spectrum defined in Eq. (S27) does not introduce a new interference mechanism beyond that present in the linear response. Instead, it reflects an intensity-dependent reweighting of the same plasmon–exciton interference channels responsible for the linear Fano lineshape. By selectively suppressing strongly absorbing resonances through population saturation, the nonlinear differential spectrum enhances the contrast of the underlying Fano interference, while preserving its characteristic phase-dependent asymmetry. Particularly, when the plasmonic resonance is tuned across the molecular resonances, the phase character of the plasmon-induced polarization reverses, thereby flipping the sequence of constructive and destructive interference for different excitonic modes. As a result, the extracted Fano parameters in the nonlinear spectra directly encode the relative phase between plasmonic and molecular responses.

In this way, the contribution of saturable absorption is clearly separated from the linear hybrid response, allowing quantitative analysis of how population dynamics shape the nonlinear optical spectrum. This framework allows us to describe the nonlinear optical response of the plasmon–exciton hybrid system, bridging the linear regime (dominated by independent resonances) and the nonlinear regime (governed by population depletion and saturation effects).

For an isolated component, the saturation intensity can in principle be determined from power-dependent nonlinear absorption measurements. However, in the plasmon–exciton hybrid structure considered here, molecules do not experience the incident field directly but rather a plasmon-enhanced local field. Consequently, the effective saturation scale becomes configuration dependent and cannot be interpreted as a fixed intrinsic material constant.

In the present semiclassical treatment, the molecular saturation intensities are introduced following the scaling relation discussed above in order to preserve the relative hierarchy among different excitonic resonances. The absolute values are therefore not regarded as independently validated parameters, and the simulated nonlinear spectra are normalized to emphasize the relative reweighting of interference channels.

Although plasmon-related nonlinear signals may appear at specific spectral positions, incorporating plasmon saturation into an ensemble-averaged full-spectrum model would introduce additional non-unique parameters associated with spatial field inhomogeneity. For clarity of the mechanism analysis, the plasmonic channel is therefore treated as effectively unsaturated in the full-spectrum simulations. This modeling choice does not affect the central conclusion that the observed nonlinear spectra originate from molecular population depletion under plasmon-enhanced excitation.

We note that the present semiclassical framework is formulated in the basis of bare plasmonic and excitonic modes, with nonlinear absorption introduced through population saturation of these modes. This description is appropriate in the weak-to-intermediate coupling regime, where plasmon–exciton interactions give rise to Fano interference but do not produce well-resolved Rabi splitting. In the strong-coupling regime, where the system eigenstates become hybrid polaritonic modes, a saturation mechanism defined in the bare-mode basis would no longer be adequate and a different theoretical treatment would be required.

**Supplementary Note 7. References**

1.	Johnson, P. B. & Christy, R. W. Optical constants of the noble metals. *Physical Review B* **6**, 4370-4379 (1972).

2.	Yang, D. J. Direct detection of ultraweak CO signal with cavity plasmon by resonant vibration–plasmon coupling. *Advanced Theory and Simulations.* **3**, 2000146 (2020).

3.	Scheffler, M. The influence of lateral interactions on the vibrational spectrum of adsorbed CO. *Surface Science* **81**, 562-570 (1979).

4.	Törmä, P. & Barnes, W. L. Strong coupling between surface plasmon polaritons and emitters: a review. *Reports on Progress in Physics* **78**, 013901 (2015).

5.	Liu, R. M. et al. Strong light-matter interactions in single open plasmonic nanocavities at the quantum optics limit. *Physical* *Review* *Letters* **118**, 237401 (2017).

6.	Li, Y. M. et al. Plasmon–exciton strong coupling in colloidal Au nanocubes with layered molecular J-aggregates. *Nano Letters* **24**, 16115-16123 (2024).

7.	Savasta, S. et al. Nanopolaritons: vacuum Rabi splitting with a single quantum dot in the center of a dimer nanoantenna. *ACS Nano* **4**, 6369-6376 (2010).

8.	Faucheaux, J. A., Fu, J. Y., Jain, P. K. Unified theoretical framework for realizing diverse regimes of strong coupling between plasmons and electronic transitions. *The* *Journal* *of* *Physical* *Chemistry* *C* **118**, 2710-2717 (2014).

9.	Zengin, G. et al. Realizing strong light-matter interactions between single-nanoparticle plasmons and molecular excitons at ambient conditions. *Physical Review Letters* **114**, 157401 (2015).

10.	Farr, E. P. et al. Introduction to time-resolved spectroscopy: nanosecond transient absorption and time-resolved fluorescence of eosin B. *Journal of Chemical Education* **95**, 864−871 (2018).

11.	Sheik-Bahae, M. et al. Sensitive measurement of optical nonlinearities using a single beam. *IEEE Journal of Quantum Electronics* **26**, 760-769 (1990).

12.	Qiu, Y. H. et al. Size-dependent plasmon relaxation dynamics and saturable absorption in gold nanorods. *Journal of Physics D: Applied Physics* **49**, 185107 (2016).

13.	Philip, R. & Kumar G. R. Picosecond optical nonlinearity in monolayer-protected gold, silver, and gold-silver alloy nanoclusters. *Physical Review B* **62**, 13160-13166 (2000).

14.	Philip, R. et al. Evolution of nonlinear optical properties: from gold atomic clusters to plasmonic nanocrystals. *Nano Letters* **12**, 4661-4667 (2012).

15.	Fano, U. Effects of configuration interaction on intensities and phase shifts. *Physical* *Review* **124**, 1866-1878 (1961).
